# A Dynamic Loop in Halohydrin Dehalogenase HheG Regulates Activity and Enantioselectivity in Epoxide Ring Opening

**DOI:** 10.1021/acscatal.4c04815

**Published:** 2024-10-14

**Authors:** Marcel Staar, Lina Ahlborn, Miquel Estévez-Gay, Katharina Pallasch, Sílvia Osuna, Anett Schallmey

**Affiliations:** † Institute for Biochemistry, Biotechnology and Bioinformatics, Technische Universität Braunschweig, Spielmannstr. 7, 38106 Braunschweig, Germany; ‡ CompBioLab Group, Institut de Química Computacional i Catàlisi (IQCC), Departament de Química, Universitat de Girona, c/Maria Aurèlia Capmany 69, 17003 Girona, Catalonia, Spain; § ICREA, Passeig Lluís Companys 23, 08010 Barcelona, Catalonia, Spain; ∥ Zentrum für Pharmaverfahrenstechnik (PVZ), Technische Universität Braunschweig, Franz-Liszt-Str. 35a, 38106 Braunschweig, Germany; ⊥ Braunschweig Integrated Center of Systems Biology (BRICS), Technische Universität Braunschweig, Rebenring 56, 38106 Braunschweig, Germany

**Keywords:** halohydrin dehalogenase, protein engineering, enantioselectivity, epoxide ring opening, molecular dynamics, cross-linked enzyme crystals

## Abstract

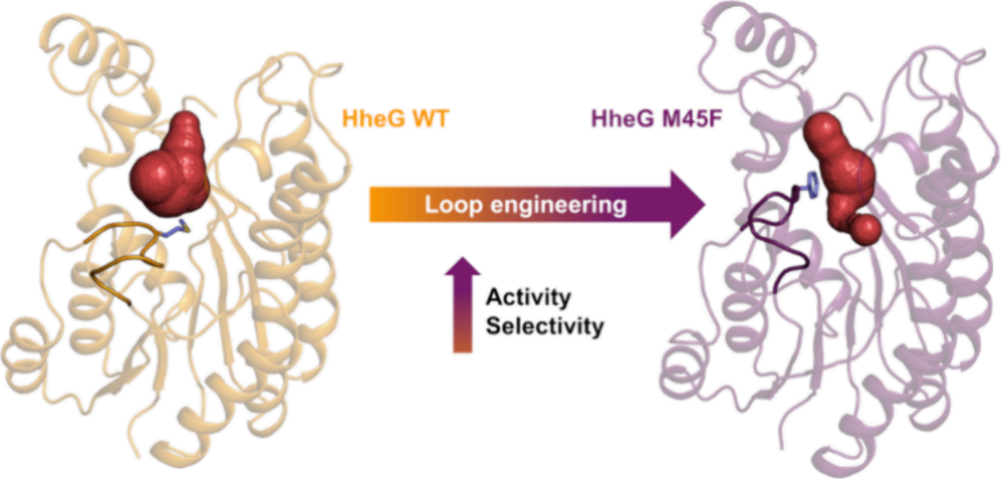

Halohydrin dehalogenase HheG and its homologues are remarkable enzymes for the efficient ring opening of sterically demanding internal epoxides using a variety of nucleophiles. The enantioselectivity of the respective wild-type enzymes, however, is usually insufficient for application and frequently requires improvement by protein engineering. We herein demonstrate that the highly flexible N-terminal loop of HheG, comprising residues 39 to 47, has a tremendous impact on the activity as well as enantioselectivity of this enzyme in the ring opening of structurally diverse epoxide substrates. Thus, highly active and enantioselective HheG variants could be accessed through targeted engineering of this loop. In this regard, variant M45F displayed almost 10-fold higher specific activity than wild type in the azidolysis of cyclohexene oxide, yielding the corresponding product (1*S*,2*S*)-2-azidocyclohexan-1-ol in 96%ee_P_ (in comparison to 49%ee_P_ for HheG wild type). Moreover, this variant was also improved regarding activity and enantioselectivity in the ring opening of cyclohexene oxide with other nucleophiles, demonstrating even inverted enantioselectivity with cyanide and cyanate. In contrast, a complete loop deletion yielded an inactive enzyme. Concomitant computational analyses of HheG M45F in comparison to wild type enzyme revealed that mutation M45F promotes the productive binding of cyclohexene oxide and azide in the active site by establishing noncovalent C–H ··π interactions between epoxide and F45. These interactions further position one of the two carbon atoms of the epoxide ring closer to the azide, resulting in higher enantioselectivity. Additionally, stable and enantioselective cross-linked enzyme crystals of HheG M45F were successfully generated after combination with mutation D114C. Overall, our study highlights that a highly flexible loop in HheG governs the enzyme’s activity and selectivity in epoxide ring opening and should thus be considered in future protein engineering campaigns of HheG.

## Introduction

Enantioselectivity is often a major driver for the application of enzyme-catalyzed reactions in chemical synthesis. Although a number of native enzymes have been described that generally display high to absolute enantioselectivity in their catalyzed reactions,^1−5^ in the majority of cases the selectivity of an enzyme varies from substrate to substrate. For this reason, protein engineering is often vital to adjust the enantioselectivity of a native enzyme (along with other enzyme characteristics) to fulfill the requirements of an industrial biocatalytic process.^6−9^

**Figure 1:**
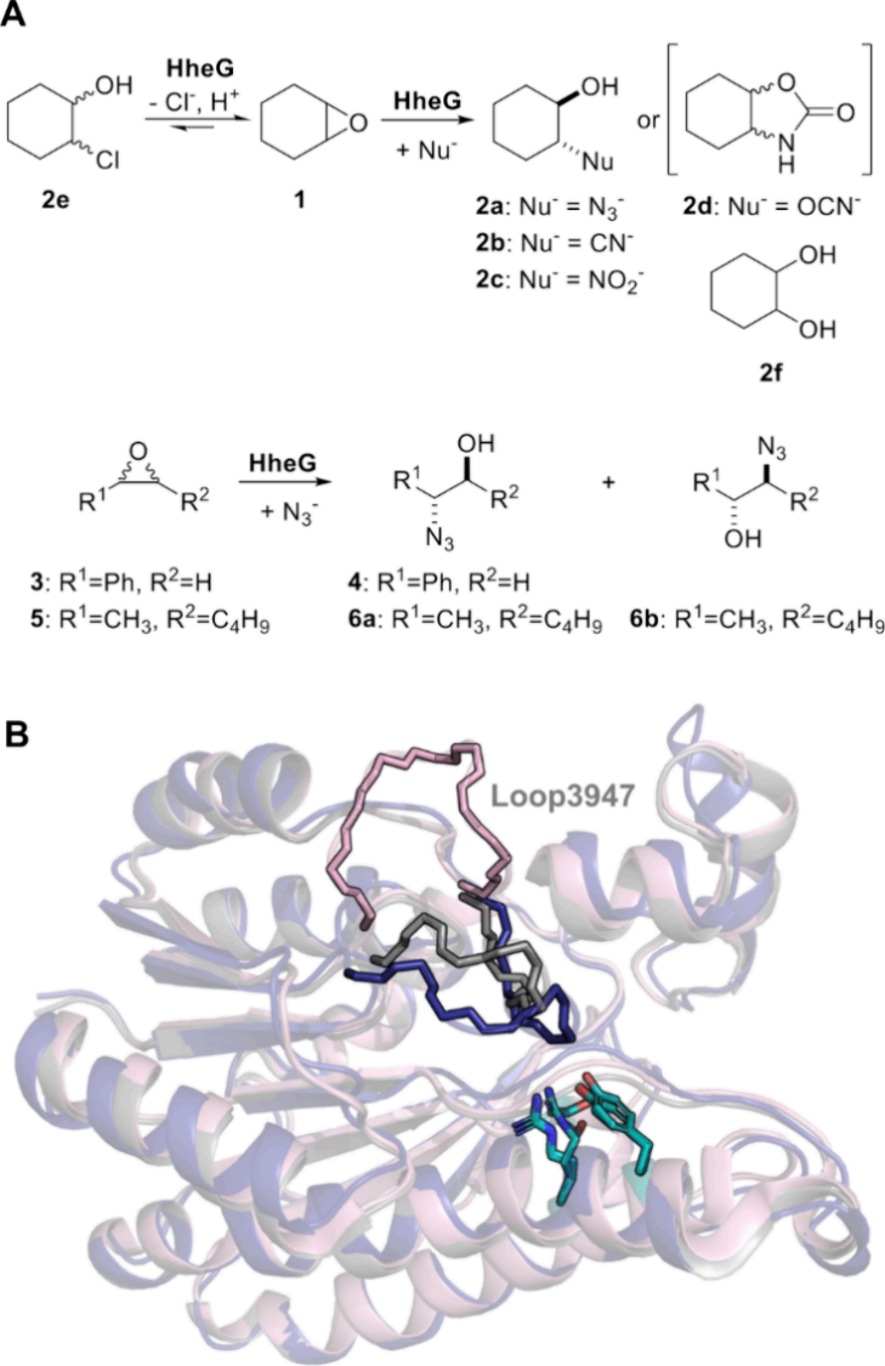
(A) Dehalogenation and epoxide ring opening reactions of the HheG wild type and its mutants performed in this study [**1**: cyclohexene oxide, **2a**: 2-azidocyclohexan-1-ol, **2b**: 2-cyanocyclohexan-1-ol, **2c**: 2-nitrocyclohexan-1-ol, **2d**: hexahydrobenzo[d]oxazol-2(3H)-one, **2e**: 2-chlorocyclohexan-1-ol, **2f**: cyclohexan-1,2-diol, **3**: styrene oxide, **4**: 2-azidophenylethan-1-ol, **5**: trans-2,3-heptene oxide, **6a**: 2-azidoheptan-3-ol, **6b**: 3-azidoheptan-2-ol]. (B) Superimposition of the crystal structure of wild-type HheG monomer (light pink; PDB: 5o30), exhibiting an open loop3947 conformation, with a simulated structure of HheG wild type (deep blue) and a crystal structure of HheG T123G monomer (gray; PDB: 6i9v), both featuring a closed loop3947 conformation. The catalytic residues of HheG (S152, Y165, R169) are highlighted in teal.

Halohydrin dehalogenases (HHDHs) have gained increasing attention in recent years for their application in the synthesis of a plethora of valuable products.^10^ Naturally, those enzymes catalyze the reversible dehalogenation of β-haloalcohols with formation of the corresponding epoxides.^11^ In the reverse reaction, a range of small *C*-, *O*-, *N*-, and *S*-nucleophiles, such as azide, cyanide, nitrite, cyanate, and thiocyanate (known as pseudohalogens), are also accepted for epoxide ring opening in addition to halide ions (see also Figure 1A).^12^ This enabled the preparation of enantioenriched β-nitroalcohols,^13^ β-cyanohydrins,^14^ oxazolidinones,^15^ and thiiranes^16^ among others,^17−20^ fueled by the recent discovery of many new HHDHs from public sequence databases.^13,21−24^ In most of these cases, however, protein engineering of the initially identified enzymes with the highest activity was still necessary to achieve also high enantioselectivity. Apart from a few examples,^13,19,25−28^ most native HHDHs do not display high enantioselectivity as this would contradict their assumed natural function, which is presumably the detoxification and degradation of (naturally occurring) haloalcohols.^29,30^

Many examples for the protein engineering of selected HHDHs, such as HheC from *Agrobacterium radiobacter* AD1, have been reported in literature that focus on enantioselectivity but also on activity and stability.^31−38^ Of these examples, the majority profited from the previously solved crystal structures of respective enzymes. Thus, crystal structures for members of almost all currently known HHDH subtypes A-G have been reported by now.^13,39−43^ One of them is HheG from *Ilumatobacter coccineus*.^41^ This enzyme was the first reported HHDH displaying unexpectedly high activity on cyclic as well as other sterically demanding internal epoxide substrates, owing to its much broader and more solvent-exposed active site compared to other HHDHs.^41,44−46^ Since its discovery in 2017, a few more G-type HHDHs have been characterized, all displaying substrate scopes as HheG.^13,46,47^ Despite this appealing activity toward bulky epoxides, HheG as well as other G-type members still come with some limitations including their insufficient stability for industrial application as well as an often only moderate enantioselectivity.^36,41,46^ The first challenge, the poor (thermal) stability of HheG, was already addressed by us through protein engineering as well as immobilization.^36,48,49^ Thus, we could demonstrate that the exchange of residue T123 in HheG by aromatic amino acids or glycine resulted in variants with up to 14 K higher apparent melting temperature as well as increased activity, most likely through regulating the dynamics of an N-terminal loop spanning residues 39–47 (Figure 1B).^36^ Interestingly, a slight increase in the enantioselectivity of those HheG variants was observed, as well. In a complementary approach to stabilize HheG for application, we recently prepared cross-linked enzyme crystals (CLECs) after crystal contact engineering of HheG to introduce defined cross-linking sites.^48,49^ This yielded highly stable and reusable enzyme preparations with remarkable resistance toward temperature, pH and the presence of organic solvents. Interestingly, some of the HheG mutants generated during crystal contact engineering also displayed improved enantioselectivity.^48,49^ This time, respective mutations (M45C and V46K) were directly located on the flexible N-terminal loop, opposite the catalytic triad of HheG (Figure 1B). This loop can occur in an open as well as closed conformation (Figure 1B), as demonstrated by X-ray analysis and MD simulations, thus confining the active site and impacting substrate access.^36,50^ Moreover, this loop is present in not only HheG from *I. coccineus* but also many other homologous G-type halohydrin dehalogenases (see Figure S1).

Very recently, the first studies on the enantioselectivity engineering of HheG have also been reported. These yielded HheG mutants with high enantioselectivity in the synthesis of chiral aryl- and spiro-oxazolidinones as well as the azidolysis of cyclohexene oxide and cyclopentene oxide.^51−54^ In all cases, substrate docking based on the published crystal structure of HheG^41^ was used to identify relevant residues within the enzyme active site for subsequent site-saturation mutagenesis. This way, even mutants displaying inverted enantioselectivity compared to HheG wild type could be identified,^52−54^ while the flexible N-terminal loop of HheG was not touched during mutagenesis. The latter is likely explained by the fact that in the crystal structure of HheG wild type (PDB: 5o30)^41^ the flexible loop is in an open conformation, thus pointing away from the active site (Figure 1B). Only for the crystal structure of the HheG mutant T123G (PDB: 6i9v), a closed loop conformation has been reported so far.^36^

As our preliminary data hinted at a potential impact of the flexible loop covering residues T39 to G47 (in the following abbreviated loop3947) on the enantioselectivity of HheG, we hypothesized that this loop might have a larger impact on the catalytic performance of HheG than previously anticipated and surmised that highly enantioselective HheG variants could also be accessed through systematic engineering of this loop. To this end, two libraries containing defined single point mutants were generated and screened in ring opening reactions of chemically diverse epoxide substrates in combination with different nucleophiles. This way, several HheG variants with largely increased or even inverted enantioselectivity as well as considerably improved activity toward the tested substrates could be identified.

## Results and Discussion

### Loop Engineering and Library Screening

**Figure 2:**
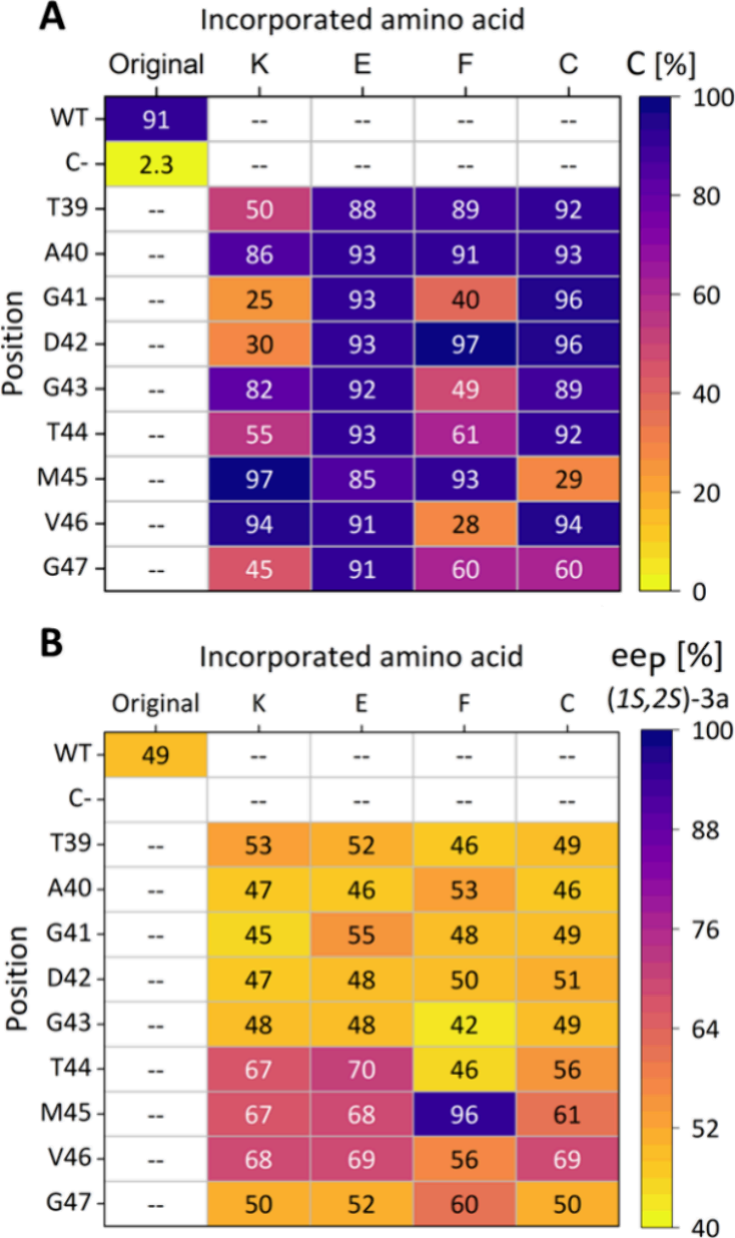
Screening result of a reduced library at loop3947 of HheG in the azidolysis of cyclohexene oxide (**1**). (A) Conversion (C), (B) product enantiomeric excess (ee_P_). Reactions were performed at 22 °C and 900 rpm in 1 mL 50 mM Tris·SO_4_, pH 7.0 using 200 μL cell-free extract (CFE), 20 mM cyclohexene oxide (**1**) and 40 mM sodium azide. Samples were taken after 2 h and analyzed by achiral and chiral GC.

As single point mutations on loop3947 of HheG turned out to influence the enantioselectivity of this enzyme, we set out to explore the impact of this loop in more detail by means of protein engineering. To minimize our screening effort, positions T39 to G47 were initially only replaced by lysine, phenylalanine, cysteine, and glutamate to incorporate a set of chemically diverse amino acids. Resulting HheG variants including wild type as well as a negative control (i.e., *E. coli* harboring an empty pET28a(+) vector) were produced in a 96-deep well plate yielding sufficient amounts of soluble enzyme (see Figure S2). This reduced library (in the form of cell-free extract, CFE) was then screened in the azidolysis of cyclohexene oxide (**1**) as a model reaction (Figure 1A), achieving moderate to high conversions with all variants within 2 h of reaction (Figure 2A). Interestingly, the corresponding ee_P_ values revealed that enantioselectivity of HheG was mainly affected by mutations at loop positions 44–46. Thus, nearly all tested variants at those three positions displayed improvements in enantioselectivity compared to HheG wild type (Figure 2B).^36,48,49^ HheG M45F even formed azidoalcohol (1*S*,2*S*)-**2a** with a product enantiomeric excess of 96%. It should be noted that the substrate cyclohexene oxide (**1**) is achiral, and only upon epoxide ring opening by a nucleophile a chiral product is formed. In this case, product chirality is determined by the regioselectivity of the nucleophilic attack at either C1 or C2 of the epoxide ring, which in turn is governed by the relative positioning of epoxide and nucleophile within the enzyme active site during catalysis. For simplicity, however, we are still using the term enantioselectivity also in case of cyclohexene oxide reactions. Moreover, a nucleophilic attack at a chiral carbon atom (as e.g. in substrates styrene oxide (**3**) and *trans*-2,3-heptene oxide (**5**), Figure 1B) proceeds with inversion of configuration in accordance with the S_N_2-type mechanism of the HHDH-catalyzed epoxide ring opening reaction.^55^

**Figure 3:**
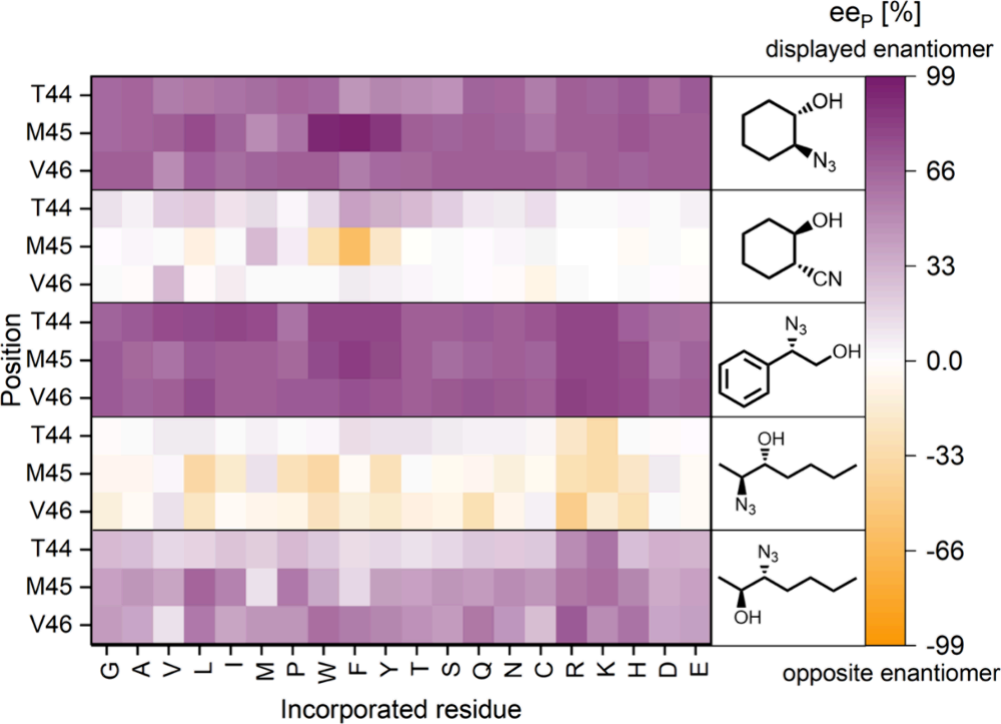
Screening result (product enantiomeric excess, ee_P_) of the fully randomized library at positions 44, 45, and 46 of HheG in ring opening reactions of cyclohexene oxide (**1**), styrene oxide (**3**) and *trans*-2,3-heptene oxide (**5**) with azide as nucleophile as well as the ring opening of **1** with cyanide. Reactions were performed at 22 °C and 900 rpm in 1 mL 50 mM Tris·SO_4_, pH 7.0 using either 100 μL (in reactions with **3** and **5**) or 200 μL CFE (in reactions with **1**), 10 or 20 mM epoxide (**1** and **3**: 20 mM; **5**: 10 mM) and 2 eq sodium azide or cyanide. Samples were taken after 2 h (azide) or 24 h (cyanide) for reactions with **1**, after 10 min in the case of reactions with **3** and after 30 min for reactions with **5**. Samples were extracted and analyzed by achiral and chiral GC. Corresponding absolute ee_P_ values as well as conversion data and *E* values are given in Tables S1–S6 in the Supporting Information.

As positions T44, M45, and V46 turned out to exert the highest impact on HheG selectivity, completely randomized libraries only on those positions were investigated next. To this end, all possible single variants were generated separately, produced in a 96-deep well plate, and screened again in the ring opening of cyclohexene oxide (**1**) with azide (again using CFE). Indeed, almost all variants displayed improved enantioselectivity compared to wild-type HheG (Figure 3), while conversion was always high (see Table S1 for absolute values). This result is in line with our reduced library data, confirming the importance of these three positions for HheG enantioselectivity. Interestingly, variants carrying an aromatic residue at position 45 yielded highest product enantiomeric excesses of 86% (M45Y), 92% (M45W), and 96% (M45F) (Table S1). This is striking as also aromatic residues at position 123 of HheG had the highest impact on the enzyme’s stability and positively influenced enantioselectivity as well, presumably through regulation of the loop3947 dynamics.^36^

To investigate whether this positive impact on HheG enantioselectivity holds true for other reactions as well, the fully randomized library at positions 44–46 was further screened in the azidolysis of styrene oxide (**3**) and *trans*-2,3-heptene oxide (**5**) as well as the ring-opening of cyclohexene oxide (**1**) with cyanide (Figures 1A and 3). With cyanide as nucleophile, conversions of all HheG variants were much smaller compared to azide (Table S2), in agreement with previous reports.^41,46^ Remarkably, variant M45F again displayed higher enantioselectivity, this time, however, for the opposite cyanoalcohol enantiomer compared to wild-type HheG,^41^ as well as a three-times higher conversion (Table S2). Also other aromatic residues at position 45 resulted in preferential formation of the (*1S,2R*)-product enantiomer, while HheG variants T44F and T44Y displayed slightly increased enantioselectivity compared to the wild type for production of (*1R,2S*)-2-cyanocyclohexan-1-ol (Figure 3 and Table S2). Thus, only a minor difference in positioning of the aromatic residue on loop3947 determines whether one or the other product enantiomer is formed, at least in the conversion of **1** with cyanide. Inversion in enantioselectivity upon loop engineering has also been reported for other HHDHs, e.g. for HheC from *Agrobacterium radiobacter* AD1 and HheA from *Arthrobacter* sp. strain AD2 in the dehalogenation of 2-chloro-1-phenylethanol.^35,56^ Both enzymes, however, do not possess such a highly flexible loop close to the N-terminus that would correspond to loop3947 of HheG.

With styrene oxide (**3**) as substrate, HheG preferentially catalyzes nucleophilic attack at the benzylic α-position (Figure 1A), which is the same as in the nonenzymatic reaction but opposite to the regioselectivity of most other HHDHs.^44^ Thus, to preclude a reduction in product enantiomeric excess during azidolysis of **3** due to the chemical background, screening reactions with **3** and azide were already stopped after 10 min, attaining between 50 and 60% conversion (Table S3). This time, several loop variants displayed an increase in enantioselectivity with preferential formation of (*S*)-2-azidophenylethan-1-ol (**4**) like the HheG wild type (Figure 3), while the highest *E*-values of 40 and 46 were observed for variants carrying a phenylalanine at position 44 or 45, respectively, corresponding roughly to a 2-fold improvement in comparison to wild-type HheG (Table S3). Hence, mutation M45F in HheG does not only impact the enzyme’s enantioselectivity in the conversion of cyclohexene oxide, but also in the transformation of a structurally unrelated epoxide like styrene oxide.

Azidolysis of the internal epoxide **5** by HheG results in the formation of two different regioisomers, 2-azidoheptan-3-ol (**6a**) and 3-azidoheptan-2-ol (**6b**) (Figure 1B).^45^ Both are roughly produced in equal amounts by wild-type HheG and this ratio did not significantly change upon loop engineering (Table S4). For several HheG variants, however, an inversion in enantiopreference compared to the wild-type enzyme for formation of regioisomer **6a** could be observed (Figure 3), even though absolute E values were still quite low (Table S5). In contrast, the enantiopreference of the loop variants in the formation of regioisomer **6b** was the same as for HheG wild type (Figure 3), but the enantioselectivity of many variants increased reaching ee_P_ values of up to 70% (for comparison, the ee_P_ of **6b** in the wild-type control was only 12%) (Table S6). This time, HheG variants T44K, M45L, M45K, and V46R turned out to yield the highest selectivity improvements, while HheG M45F displayed only minor changes in enantioselectivity compared to wild type (Figure 3 and Tables S5 and S6).

In summary, screening of the fully randomized library at positions 44–46 with structurally different substrates and nucleophiles always revealed several amino acid exchanges exerting a strong impact on HheG’s enantioselectivity. This confirms our initial hypothesis that more selective HheG variants can indeed be accessed through the engineering of loop3947. Moreover, only one mutation (M45F) on loop3947 was sufficient to obtain a highly selective HheG mutant for the azidolysis of cyclohexene oxide (**1**), giving azidoalcohol (1*S*,2*S*)-**2a** with 96%ee. In contrast, in the work by Tian et al.^53^ based on the structure-guided mutagenesis of active-site residues of HheG, a triple mutant (Y18G/M189L/F200W) was necessary to obtain the same product enantiomer with 94%ee. This further emphasizes the importance of loop3947 for the enantioselectivity of HheG.

### Characterization of Beneficial Variants

To validate and further characterize the most beneficial HheG variants observed during screening, respective enzymes were produced on a 100 mL scale and purified via immobilized metal ion affinity chromatography (IMAC). Subsequently, their specific activities and selectivities in respective epoxide ring opening reactions, for which they had been identified during screening, as well as their apparent melting temperatures were determined (Tables 1 and S7). Additionally, ring opening of **1** by HheG variants M45F, M45Y, and M45W was also investigated using cyanate and nitrite, as HheG was recently shown to accept a broader range of nucleophiles.^46^

**Table 1:** Characterization Data of Purified HheG Variants in Epoxide Ring Opening Reactions using Different Nucleophiles^a^

substrate	HheG variant	conversion (%)		product enantiomeric excess (%)		*E* value	
**1** + N_3_^–^	WT	71 ± 0.2^b^		49 ± 0.4% (*1S,2S*)^b^			
	M45F	99 ± 0.1^b^		96 ± 0.8% (*1S,2S*)^b^			
	M45Y	97 ± 2.0^b^		86 ± 0.3% (*1S,2S*)^b^			
	M45W	79 ± 9.3^b^		91 ± 0.1% (*1S,2S*)^b^			
**1** + CN^–^	WT	20 ± 0.5%^c^		28 ± 0.0% (*1R*,*2S*)^c^			
	M45F	57 ± 0.3%^c^		60 ± 0.1% (*1S,2R*)^c^			
	M45Y	13 ± 1.4%^c^		19 ± 0.9% (*1S,2R*)^c^			
	M45W	10 ± 0.7%^c^		24 ± 0.9% (*1S,2R*)^c^			
**1** + OCN^–^	WT	54 ± 0.9%^c^		57 ± 0.3%^c^^,^^d^			
	M45F	95 ± 0.6%^c^		–40 ± 0.1%^c^^,^^d^			
	M45Y	18 ± 0.5%^c^		1.8 ± 0.0%^c^^,^^d^			
	M45W	7.2 ± 0.0%^c^		–30 ± 0.0%^c^^,^^d^			
**1** + NO_2_^–^	WT	15 ± 0.1%^c^ (73:27)^e^		0.1 ± 0.1%^c^^,^^d^			
	M45F	87 ± 0.0%^c^ (60:40)^e^		44 ± 0.6%^c^^,^^d^			
	M45Y	10 ± 0.1%^c^ (68:32)^e^		5.4 ± 0.3%^c^^,^^d^			
	M45W	9.3 ± 0.2%^c^ (65:35)^e^		3.1 ± 0.3%^c^^,^^d^			
**3** + N_3_^–^	WT	46 ± 1.4%^f^		84 ± 0.4% (*2S*)^f^		24 ± 1.9	
	T44F	53 ± 0.8%^f^		84 ± 1.0% (*2S*)^f^		44 ± 1.6	
	T44Y	53 ± 0.6%^f^		85 ± 0.4% (*2S*)^f^		42 ± 0.4	
	T44W	52 ± 0.8%^f^		82 ± 1.1% (*2S*)^f^		32 ± 0.4	
	M45F	52 ± 0.0%^f^		85 ± 0.1% (*2S*)^f^		39 ± 0.2	
**5** + N_3_^–^		**6a**	**6b**	**6a**	**6b**	**6a**	**6b**
	WT	48 ± 0.1%^g^	51 ± 0.1%^g^	12 ± 0.3% (*2S,3R*)^g^	12 ± 0.0% (*2S,3R*)^g^	1.4 ± 0.0	1.4 ± 0.0
	T44K	36 ± 0.2%^g^	40 ± 0.1%^g^	4.7 ± 1.7% (*2R,3S*)^g^	38 ± 1.2% (*2S,3R*)^g^	1.1 ± 0.1	2.8 ± 0.1
	M45L	33 ± 0.4%^g^	45 ± 0.5%^g^	5.6 ± 0.6% (*2S,3R*)^g^	36 ± 0.6% (*2S,3R*)^g^	1.1 ± 0.0	2.8 ± 0.0
	M45K	25 ± 0.2%^g^	30 ± 0.2%^g^	29 ± 0.6% (*2R,3S*)^g^	61 ± 0.4% (*2S,3R*)^g^	2.0 ± 0.0	5.3 ± 0.1
	V46R	32 ± 1.0%^g^	36 ± 0.9%^g^	10 ± 2.8% (*2R,3S*)^g^	46 ± 1.9% (*2S,3R*)^g^	1.3 ± 0.1	3.5 ± 0.2

a Reactions were performed at 22 °C in 50 mM Tris·SO_4_ buffer, pH 7.0, for the indicated amount of time and analyzed by achiral and chiral GC. All reactions were performed in duplicate.

b Determined after 2 h.

c Determined after 24 h.

d Enantiomers unassigned.

e Product ratio of nitroalcohol **2c**:diol **2f**.

f Determined after 10 min.

g Determined after 3 h.

The resulting data do not only confirm the improvements in enantioselectivity observed during screening, but also highlight a concomitant increase in activity upon mutagenesis, which is especially evident for HheG M45F. This variant did not only achieve higher conversions compared to wild type in all studied epoxide ring opening reactions (Table 1), but displayed also an almost 10-fold higher specific activity in the azidolysis of **1** (Table S7), as determined by our recently published BTB assay.^57^ Moreover, variant M45F was more than twice as active as HheG wild type in the ring opening of **3** with azide, while variant T44F exhibited the highest specific activity (Table S7) and the highest enantioselectivity (*E* = 44, Table 1) in this reaction. Compared to other literature reports, this *E* value of 44 is not as high as that reported for other HHDHs, e.g., HheC^18^ or HheA2 N178A,^35^ that—unlike HheG—display β-regioselectivity in the ring opening of **3**. In contrast to most other HHDHs with α-regioselectivity,^13,47^ however, all HheG variants preferentially convert (*R*)-styrene oxide yielding azidoalcohol (*S*)-**4** as product.

When looking at the ring opening of epoxide **1** with cyanate and nitrite, the selectivity of variant M45F was again altered significantly in comparison to HheG wild type (Table 1). Using cyanate as a nucleophile, M45F displayed an inverted enantioselectivity, as also observed with cyanide, while activity was increased as well. In the reaction with nitrite, only HheG variant M45F exhibited considerably enhanced selectivity and activity compared to HheG wild type, while the ratio of formed nitroalcohol:diol (**2c**:**2f**) was slightly affected for all tested variants (Table 1). Interestingly, HheG M45F was not only more active in epoxide ring opening reactions but also exhibited a higher specific activity in the dehalogenation of 2-chlorocyclohexan-1-ol (**2e**) (0.07 U mg^–1^ for variant M45F compared to 0.02 U mg^–1^ for HheG wild type). Overall, our data using purified variants highlight that loop3947 in HheG does not only play a central role for the selectivity of HheG but also its activity. In contrast, the thermal stability of all tested variants seems to be hardly affected upon engineering of loop3947, as the determined apparent melting temperatures varied only slightly between the variants and HheG wild type (Table S7). Previously, we reported that aromatic amino acids as well as glycine at position 123 resulted in significantly thermostable HheG variants.^36^ Moreover, a possible interaction between positions M45 and T123 was hypothesized.^36^ The combination of beneficial mutations at position 45 with respective mutations at position 123 indeed yielded thermostabilized variants (exhibiting up to 12 K increase in apparent melting temperature, Table S7) that retained a high selectivity and, in some cases, were even further improved in terms of activity (see Tables S7 and S8). Likewise, a combination of the identified beneficial loop mutations with, e.g., mutations at active-site residues of HheG could be carried out to increase also the enantioselectivity further.

Regarding the ring opening of epoxide **5** with azide, the inversion in enantioselectivity for the formation of regioisomer **6a** as well as the improvement in enantioselectivity for the generation of regioisomer **6b** of the purified variants in comparison to wild-type HheG could be confirmed, while absolute E-values of the selected variants were lower compared to our initial screening results (Table 1). The latter is probably the result of the higher conversions (between 30 and 50%) that we aimed for in our reactions using purified enzymes in comparison to the screening. Based on these results, positions 44–46 on the loop are likely not the only relevant residues in HheG to steer the enzyme’s enantioselectivity in the azidolysis of epoxide **5**.

To better understand why mutant M45F also displayed a tremendous increase in activity compared to that of the HheG wild type, kinetic parameters of this variant in the azidolysis of cyclohexene oxide (**1**) were determined using our BTB assay (Table 2). This revealed not only a 5–15-fold increase in maximal reaction velocity (*k*_obs,max_) of mutant M45F (rate improvement varies when either the kinetics for epoxide or azide are considered) but also a significant improvement in azide binding compared to HheG wild type. Interestingly, this loop mutation seems to enhance the cooperativity for azide binding, as the respective Hill coefficient *n* increased as well. A recent conformational landscape analysis of HheG demonstrated that loop3947 would affect the presence and shape of substrate tunnel T3 in HheG.^50^ The latter might impact nucleophile binding in HheG as well.

**Table 2:** Kinetic Parameters of HheG Wild Type and Mutant M45F in the Azidolysis of Cyclohexene Oxide (**1**)^a^

HheG variant	cyclohexene oxide				azide			
	*K*_50_ (mM)	*k*_obs,max_ (s^–1^)	*k*_obs,max_/*K*_50_ (s^–1^ mM^–1^)	*n* (−)	*K*_50_ (mM)	*k*_obs,max_ (s^–1^)	*k*_obs,max_/*K*_50_ (s^–1^ mM^–1^)	*n* (−)
wild type^57^	39.4 ± 2.69	2.31 ± 0.15	0.06 ± 0.01	3.81 ± 0.78	38.4 ± 2.09	4.12 ± 0.15	0.11 ± 0.01	2.92 ± 0.36
M45F	28.0 ± 0.64	31.1 ± 0.39	1.11 ± 0.03	3.33 ± 0.27	10.1 ± 0.21	20.2 ± 0.26	1.99 ± 0.01	3.94 ± 0.29

a First, the epoxide concentration was varied while keeping the azide concentration constant at 60 mM; afterward, the azide concentration was varied while fixing the epoxide concentration at 100 mM. The Hill equation was used to fit the resulting data in OriginPro (Figure S3). Data for HheG wild type were taken from Staar et al.,^57^ as the exact same reaction conditions have been applied.

### Computational Analyses

**Figure 4:**
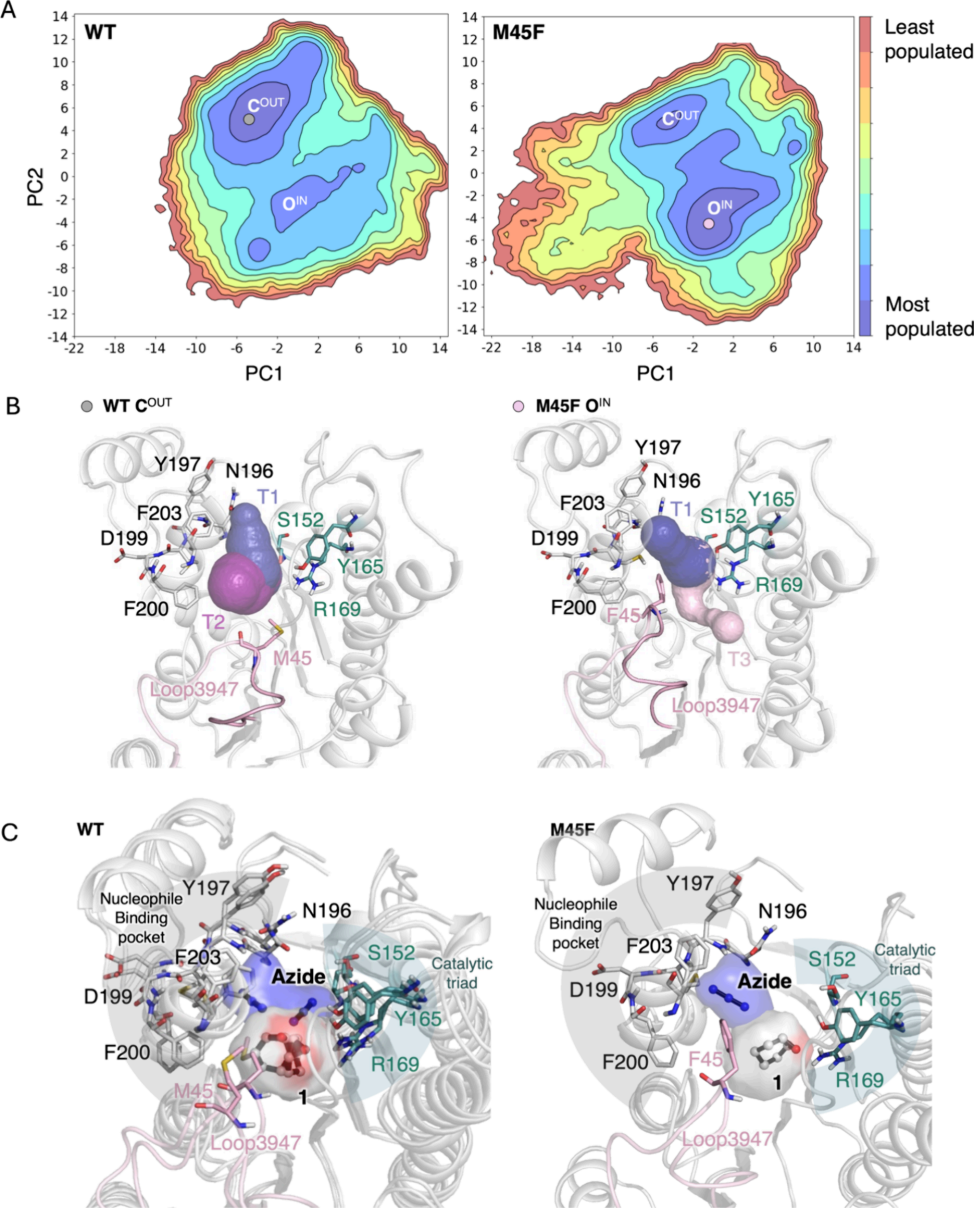
(A) Estimated free energy landscapes (FEL) of the wild type HheG (WT) and variant M45F in the absence of any substrate. PC1 and PC2 describe the open/closed conformational change of loop3947 and the side chain orientation of M/F45 within the active site pocket. Most stable conformations are colored in blue, whereas least stable ones in red. (B) Representative structure of the most populated minima is displayed together with the available tunnels: **C**^OUT^ conformation for wild type (WT) presenting the loop closed and the side chain of M45 outside the active site pocket, and **O**^IN^ of variant M45F in which loop3947 is open and F45 accommodated in the active site. Tunnel 1 (T1) is shown in dark blue, whereas tunnel 2 (T2) is in purple, and tunnel 3 (T3) is in light pink. (C) Representative structures of HheG wild type (WT) and variant M45F taken from the MD simulations performed in the presence of both cyclohexene oxide (**1**) and azide. For the WT, two different conformations of the protein are overlaid. Surface representation containing all poses of **1** and azide sampled is displayed. Cyclohexene oxide (**1**) and especially azide can adopt multiple conformations in the active site pocket of the wild type HheG, in line with its inferior catalytic activity. Nucleophile binding site residues are shown using gray sticks, catalytic residues in teal, and loop3947 and position 45 in light pink. Cyclohexene oxide (**1**) and azide are represented by using spheres and black sticks.

Intrigued by how mutation M45F enhances HheG‘s activity and enantioselectivity toward the epoxide-ring opening reaction of cyclohexene oxide (**1**) with azide, we decided to computationally evaluate HheG wild type and mutant M45F by means of Molecular Dynamics (MD) simulations in the presence and absence of substrates. M45F is contained in loop3947, whose conformational dynamics regulate the formation of the available tunnels for substrate binding and product release.^50^ We found in a previous study that the conformational flexibility of loop3947, which is located adjacent to the active site pocket, was crucial for providing HheG with the ability to accept bulkier epoxide substrates.^50^ Our study first focused on the comparison of the conformational dynamics of HheG wild type and variant M45F (Figure 4A). We performed 5 replicas of 250 ns MD simulations (1.25 μs per system in tetrameric conformation) for both systems in (i) the absence of any ligand and (ii) with both azide and epoxide **1** bound in the active site (see experimental section for computational details). We performed Principal Component Analysis (PCA) considering the pairwise distances between the heavy atoms of residues included in loop3947 and the rest of the residues of the protein. The reconstructed free energy landscapes (FELs) indicate that wild type and M45F adopt two different conformations of loop3947: a closed (**C**^OUT^) conformation in which the side chain of residue M/F45 points outside the active site, and an open conformation presenting M/F45 inside of the pocket (**O**^IN^, see Figure 4A,B). The estimated FELs suggest that in the absence of any ligand wild type mostly adopts the **C**^OUT^ conformation of loop3947, whereas in the case of M45F both conformations are visited, being **O**^IN^ the most stable minima. This different **C**^OUT^/**O**^IN^ conformation of loop3947 has a large impact on the available tunnels for substrate binding and product release. As shown in Figure 4B, the closed conformation of loop3947 favors the formation of tunnels named T1, previously found to be present in most HHDHs, and T2, which is mostly found in HheG and HheC.^50^ The open conformation of loop3947 with the side chain of M/F45 in the active site blocks the formation of tunnel 2 (T2), but instead opens tunnel 3 (T3) that is mostly observed in G-type HHDHs (Figures 4B, S4 and Table S10).^50^ The analysis of the conformational landscape and available tunnels for the HheG wild type and variant M45F suggests that the introduced mutation favors the exploration of both open and closed conformations of loop3947, which affects T2/T3 formation potentially impacting the productive binding of both substrates in the active site pocket, as well as product release.

The MD simulations performed in the presence of both substrates indicate that in wild-type HheG the side chain of M45 is highly flexible, which affects the positioning of the epoxide and azide in the active site. The reconstructed FELs in the presence of epoxide and azide (Figure S5A) show that additional minima are sampled in both wild-type HheG and mutant M45F in comparison to the reconstructed FELs without substrate, while conformation **O**^IN^ is the one presenting catalytically competent distances for the epoxide-ring opening reaction. As shown in Figure 4C, in conformation **O**^IN^ azide can be either retained in the nucleophile binding site (gray region in Figure 4C) or get displaced close to the catalytic serine (teal region in Figure 4C), which hampers the epoxide-ring opening reaction. The introduction of mutation M45F favors the productive binding of cyclohexene oxide (**1**) in the active site by establishing noncovalent C–H ··π interactions with F45 (Figure S5B). At the same time, azide is preferentially bound at the nucleophile binding pocket (gray region in Figure 4C). These simulations therefore indicate that mutation M45F helps retain both the epoxide and azide in a catalytically competent pose in the active site pocket, which is in line with the lower K_50_ for azide and the higher *k*_obs,max_ observed experimentally (Table 2).

To estimate the differences in enantioselectivity, we performed Quantum Mechanics (QM) *theozyme* calculations to determine the ideal distances and angles for the azide-mediated cyclohexene oxide ring-opening reaction and evaluated the number of MD frames displaying a pro-*S*/pro-*R* conformation. QM calculations indicate that the nucleophilic attack at the C_1_ position is favored over C_2_ by ca. 3.8 kcal/mol (Figure S6), thus indicating that the formation of the (S)-enantiomer is intrinsically favored if azide and epoxide are properly retained in the active site pocket. The nucleophilic attack distance at the transition state is ca. 2.2 Å with an N_azide_-N_azide_-C_(**1**)_ angle of ca. 109°, whereas in the reactant complex the distance is ca. 3.2 Å and the angle is 85° (Figure S5). We filtered the number of MD frames presenting catalytically competent poses by considering distances <4 Å between the epoxide oxygen of **1** and the catalytic Tyr165, as well as the distance (<4 Å) and angle (range of 80–120°) between azide and either C_1_/C_2_ of cyclohexene oxide corresponding to pro-*S*/pro-*R* attacks, respectively (see Figure S6). Using this filtering scheme, we find a higher proportion of frames presenting proper catalytic distances and angles for the pro-*S* attack in the case of the M45F variant, in line with the higher ee observed experimentally (Table S11). These simulations therefore suggest that mutation M45F restricts the flexibility of position 45 by establishing noncovalent C–H··π interactions with the epoxide. This interaction observed in M45F helps retain the substrate and azide in the active site and promotes the enantioselective epoxide-ring opening reaction by positioning C_1_ closer to the azide for the selective formation of the (1*S*,2*S*)-product.

### Loop Deletion

To gain further insights regarding the impact of the whole loop on HheG performance, HheG variants lacking either the complete loop3947 or parts of it were investigated as well. To this end, three defined variants were generated: HheG Del39–47 with complete deletion of loop3947, HheG Del44–46 where only residues T44, M45, and V46 were eliminated, and HheG Ins-DPAE in which loop3947 was replaced by a short linker containing residues aspartate, proline, alanine, and glutamate. This short sequence is present at the corresponding position of loop3947 in an HheG homologue from *Actinomycetota bacterium* (the same protein was also reported from *Acidimicrobiia bacterium*) that was recently described.^13,58^ Deletion of all residues from position 39 to 47 resulted in complete inactivation of HheG, even though the soluble, and thus folded, enzyme could still be obtained (data not shown). In contrast, elimination of residues T44, M45, V46 (HheG Del44–46) as well as exchange of loop3947 with the short linker sequence (HheG Ins-DPAE) yielded active variants displaying significantly increased specific activity in the azidolysis of **3** compared to wild-type HheG, while the activity in the ring opening of **1** was generally reduced (Table S7). The enantioselectivity of both variants was also altered compared to that of the HheG wild type, but no general trend could be observed (Table S9). Interestingly, both deletion variants again formed (1*S*,2*R*)-**2b** with slight preference in the cyanolysis of **1**, which is the opposite enantiomer to that formed by HheG wild type preferentially. This further underlines the importance of loop3947 for regulation of HheG‘s activity and enantioselectivity, probably through differences in substrate and nucleophile positioning within the active site. Remarkably, complete deletion of loop3947 (as in HheG Del39–47) considerably decreased enzyme stability as well, as evident from a decrease in the apparent melting temperature (*T*_m_) by 5 K in comparison to wild-type HheG. In contrast, corresponding *T*_m_ values of deletion variants Del44–47 and Ins-DPAE were only slightly reduced (Table S7).

### Construction of a Stable and Enantioselective Biocatalyst

Recently, we reported the efficient immobilization of HheG (especially variant D114C) in the form of cross-linked enzyme crystals (CLECs),^48,49^ which yielded an HheG preparation displaying high process stability as well as easy operability in different chemical reactor systems (batch and continuous flow).^59^ Thus, we herein aimed to combine mutation M45F with D114C with the goal of preparing enantioselective HheG CLECs. The resulting double mutant HheG M45F-D114C could be obtained in high yield (231 mg L^–1^ in comparison to 140 mg L^–1^ for HheG M45F) and displayed a similar specific activity in the azidolysis of **1** (18.3 U mg^–1^) as HheG M45F (16.5 U mg^–1^). Crystallization of this double mutant using the optimized crystallization conditions of HheG D114C led to hexagonal-shaped crystals after 24 h (Figure S7). Cross-linking with BMOE yielded stable HheG M45F-D114C CLECs that achieved 92% conversion in the ring opening of 20 mM **1** with azide already after 1 h and a high product enantiomeric excess (ee_P_) of 95% (in comparison to 82% conversion and 49%ee_P_ using wild-type HheG; Figure S8). Therefore, respective CLECs were subsequently applied in a semipreparative reaction using 50 mM cyclohexene oxide (**1**) and 2 equiv azide in 10 mL scale. After 2 h, full conversion was reached, and (1*S*,2*S*)-2-azidocyclohexan-1-ol (**2a**) was obtained in 74% isolated yield (52 mg) with an ee_P_ of 96%. This demonstrates that HheG variant M45F can be stabilized successfully via CLEC formation yielding a stable and highly enantioselective HheG preparation for future application in repetitive batch and continuous flow.

## Conclusions

Overall, we have demonstrated that loop3947 is highly important for regulating the activity as well as enantioselectivity of HheG. In this context, variant M45F was identified, displaying greatly increased activity and improved enantioselectivity in the ring opening of cyclohexene oxide with various nucleophiles. Likewise, HheG T44F displayed the highest activity and enantioselectivity increase in the azidolysis of styrene oxide among the screened loop3947 variants. In contrast, complete deletion of this dynamic loop resulted in a soluble but inactive enzyme. This highlights the significance of loop3947 for catalytic performance of HheG. As many homologues of HheG, of which only few have been characterized so far,^46^ feature comparable loops in a structurally equivalent position (see Figure S1), a similar impact of loop mutations on their catalytic performance is to be expected. Hence, future protein engineering campaigns of HheG or its homologues to improve or alter activity and enantioselectivity should not only focus on active-site mutations but also include loop variations as well. Moreover, since HHDHs are related to short-chain dehydrogenases and reductases (SDR superfamily) sharing significant homology on a sequence and structure level,^60^ our results could potentially be relevant for the engineering of those enzymes as well.

In addition, by combining the enantioselectivity-conferring mutation M45F with mutation D114C, which facilitates to immobilize HheG as CLECs,^59^ a stable biocatalyst was created exhibiting also improved activity and enantioselectivity. This does not only enhance the industrial applicability of HheG M45F, but further demonstrates that our previously published approach of HheG CLEC formation^48,49^ can be expanded to other HheG variants.

## Methods

### HheG Engineering

Protein engineering of HheG with the aim to increase its enantioselectivity focused on the exchange of residues on loop3947. In a first step, positions T39 to G47 were exchanged by amino acids Cys, Lys, Glu and Phe. Site-directed mutagenesis of HheG was performed using a PfuUltra II Hotstart PCR Mastermix (Agilent Technologies, Santa-Clara, CA, United States). Respective forward and reverse mutagenic primers (Table S12) were designed with PrimerX (Carlo Lapid, 2003, http://bioinformatics.org/primerx/index.htm, accessed on 16 February 2021), purchased from Merck (Darmstadt, Germany) and used in concentrations of 0.25 μM each with 100 ng of pET28a(+)-*hheG* template.^36^ Otherwise, the PCR protocol for mutagenesis was in agreement with the manufacturer’s instructions. Afterward, methylated parental DNA was digested at 37 °C overnight using 20 U DpnI before transformation in *E. coli* BL21(DE3) Gold.

In a second step, positions T44, M45, and V46 were fully randomized. GoldenGate cloning^61^ was used to incorporate all missing mutations at respective positions separately. The PCR using Q5 polymerase (NEB) was performed according to the manufacturer‘s instructions. Forward and reverse mutagenic primers (Table S12) were designed according to GoldenGate primer design,^61^ purchased from Merck (Darmstadt, Germany) and used in concentrations of 0.25 μM each with 5 ng of pET28a(+)-*hheG* template. Each 100 ng PCR product were purified using the E.Z.N.A. MircoElute CyclePure Kit (omega-biotek) and incubated with 2 U BsaI plus 200 U T4-Ligase in 1× CutSmart buffer and 1× T4-ligase buffer for 2 h at 30 °C. Inactivated GoldenGate reactions (20 min, 65 °C) were afterward transformed in *E. coli* BL21(DE3) Gold.

For combination of mutations M45F, M45Y, and M45W with previously described mutations T123G and T123F of HheG,^36^ site-directed mutagenesis or GoldenGate cloning was performed according to the protocols described above and using templates pET28a(+)-*hheG T123G* and pET28a(+)-*hheG T123F*. For a combination of mutations M45F and D114C in HheG, site-directed mutagenesis starting from template pET28a(+)-*hheG D114C* was used. Loop3947 deletion mutants of HheG were constructed by GoldenGate cloning using pET28a(+)-*hheG* as a template and mutagenic primers listed in Table S12.

### Protein Production in 96-Well Format

For library expression in 96-deep-well plates (HJ Bioanalytik, Erkelenz, Germany), each 1 mL of terrific broth (TB) medium (per liter: 4 mL of glycerol, 12 g of peptone, 24 g of yeast extract, 0.17 M KH_2_PO_4_, 0.74 M K_2_HPO_4_) supplemented with 50 μg mL^–1^ kanamycine and 0.2 mM isopropyl-β-thiogalactopyranosid (IPTG) was inoculated with 10% (v/v) overnight preculture. Protein production was carried out at room temperature and 1050 rpm for 24 h. Cells were harvested by centrifugation (3494 *g*, 20 min, 4 °C) and cell pellets were stored in the deep-well plate at −20 °C until further use.

Cell lysis was performed by freezing and thawing cycles. Frozen cell pellets were resuspended in each 300 μL of Tris·SO_4_ buffer, pH 7.0 supplemented with 1 mg mL^–1^ lysozyme and 1 pierce protease inhibitor tablet (Thermo Fisher Scientific) per 10 mL. The cell suspension was incubated at 30 °C and 700 rpm for 1 h before freezing at −20 °C for 30 min. The cell suspension was thawed again and incubated for another 1 h at 30 °C and 700 rpm. After the first 30 min, 50 μL of DNaseI-solution (0.1 mg mL^–1^ DNase in 20 mM MgSO_4_) was added. Afterward, the suspension was centrifuged (3494 *g*, 60 min, 4 °C) and the resulting cell free extract (CFE) was used for library screening.

### Library Screening

Library screening with different epoxides and nucleophiles was performed in glas vials using each 1 mL 50 mM Tris·SO_4_ buffer, pH 7.0 containing 200 μL CFE (100 μL in case of styrene oxide **3**) of respective library variants as well as 20 mM epoxide (10 mM in case of *trans*-2,3-heptene oxide **5**) and 2 eq of nucleophile at 22 °C and 900 rpm. Samples were taken at different time points (2 and 24 h with epoxide **1** + azide and haloalcohol **2e**; 24 h with epoxide **1**+ cyanide; 10 min with epoxide **3** + azide; 30 min with epoxide **5**+azide) and extracted with an equal volume of *tert*-buthylmethyl ether (TBME) containing 0.1% dodecane as internal standard. Organic phases were dried over MgSO_4_ and analyzed via achiral (conversion of **1** and **5**) and chiral GC (enantiomeric excesses as well as conversion of **3**). Temperature programs and retention times of substrates and products are listed in Table S13.

### BTB Assay

Specific activities of HheG variants in epoxide ring opening reactions were determined by bromothymolblue (BTB) assay as described previously.^57^ Reactions were performed in 1 mL of 2 mM MOPS buffer, pH 7.0 containing 20 mM epoxide (only 10 mM in case of epoxide **5**), 2 equiv of azide, and 5–500 μg of enzyme. Samples were taken within 4 min for styrene oxide (**3**), within 6 min for *trans*-2,3-heptene oxide (**5**) and within 15 min for cyclohexene oxide (**1**). Each 100 μL sample was mixed with an equal volume of 40 μg mL^–1^ BTB dissolved in 100% (v/v) methanol in 96-well microtiter plates (Sarstedt, Nümbrecht, Germany), and analyzed regarding absorbance at 499 and 616 nm using a CLARIOstar plate reader (BMG Labtech, Ortenberg, Germany). The subsequent calculation of consumed protons and resulting activities was performed as described previously.^57^

The BTB assay was also used to determine kinetic parameters of HheG variant M45F in epoxide ring opening of cyclohexene oxide (**1**) with azide based on initial reaction velocities. General reaction conditions were the same as for specific activity determination, but only 10 μg of HheG M45F was applied. First, the concentration of epoxide **1** was varied (5, 10, 20, 25, 30, 40, 50, 60, 80, 90, 100, 110, 120, 130, 140, and 150 mM) while keeping the azide concentration constant at 60 mM. Afterward, the azide concentration was varied (1, 2.5, 5, 7.5, 10, 15, 20, 40, 60, 80, 100 mM) while keeping the cyclohexene oxide (**1**) concentration constant at 100 mM. Reactions were performed in duplicate. To determine initial reaction velocities, each 100 μL sample was taken after 30, 60, 90, and 120 s. Resulting activities [in μmol min^–1^] were calculated as described previously,^57^ plotted over the applied substrate concentration and fitted in OriginPro 2021 using a Hill fit.

### MD Simulations

Parameters for substrates **1** and azide were generated with the antechamber and parmchk2 modules of AMBER20^62^ using the second generation of the general amber force-field (GAFF2).^63,64^ Partial charges (RESP model)^65^ were set to fit the electrostatic potential generated at the HF/6-31G(d) level of theory. The charges were calculated according to the Merz–Singh–Kollman^66,67^ scheme using the Gaussian16 software package.^68^ The protonation states were predicted using PROPKA.^69,70^ The enzyme structure was obtained from the PDB with the code 5o30^41^ and cleaned from other nonpeptidic molecules to obtain the wild-type system in a tetrameric oligomerization state. The single mutation M45F was introduced by using the Pymol mutagenesis tool. Proteins were solvated in a pre-equilibrated truncated octahedral box of 12 Å edge distance using the OPC water model, resulting in the addition of ca. 21.300 water molecules, and neutralized by the addition of explicit counterions (i.e., Na^+^) using the AMBER20 leap module. All MD simulations were performed using the amber19 force field (ff19SB)^71^ in our in-house GPU cluster, GALATEA.

The Pmemd.cuda program from Amber20 was used to perform a two-stage geometry optimization. In the first stage, solvent molecules and ions were minimized, while solute molecules were restrained using 500 kcal·mol^–1^·Å^–2^ harmonic positional restraints. In the second stage, an unrestrained minimization was performed. The systems were then gradually heated by increasing the temperature by 50 K during six 20 ps sequential MD simulations (0–300 K) under constant volume. Harmonic restraints of 10 kcal·mol^–1^·Å^–2^ were applied to the solute, and the Langevin equilibration scheme was used to control and equalize the temperature. The time step was kept at one fs during the heating stages to allow potential inhomogeneities to self-adjust. Each system was then equilibrated without restraints for 2 ns at a constant pressure of 1 atm and a temperature of 300 K using a 2 fs time step in the isothermal–isobaric ensemble (NPT). After equilibration, five replicas of 250 ns were run for each system (i.e., 1.25 μs per system and 5 μs in total simulated time) in the canonical ensemble (NVT). MD simulations were analyzed by monomers to make them easier to study, multiplying the simulated time by four. All analysis was done using available Python libraries (pyemma,^72^ mdtraj,^73^ and mdanalysis^74^) in a jupyter lab environment.

### QM Calculations

Geometry minimizations were performed using Gaussian16,^68^ using the hybrid density functional theory method B3LYP^75,76^ including D3 dispersion corrections, and the 6-31+G(d,p) basis set. Solvation effects were considered using the SMD solvation model, a variation of Truhlar’s and co-workers’ integral equation formalism variant (IEFPCM),^77^ using diethyl ether as solvent. All energies were calculated by performing single-point calculations on the optimized B3LYP-D3/6-31+G(d,p) geometries using the functional wB97XD^78^ with the 6-311+G(2d,2p) basis set.

## Supporting Information Available

Additional materials and methods descriptions and supporting figures and tables regarding computational analyses as well as performed experiments of the study (PDF)
