# Supplementary material for: A Dynamic Loop in Halohydrin Dehalogenase HheG Regulates Activity and Enantioselectivity in Epoxide Ring Opening
Source: ACS Catal. 2024 Oct 14;14(21):15976–87. doi: 10.1021/acscatal.4c04815 (PMC11536340; doi:10.1021/acscatal.4c04815)
Supplement: Supplementary file 1 — cs4c04815_si_001.pdf [file cs4c04815_si_001.pdf]

Supporting Information

A dynamic loop in halohydrin dehalogenase HheG regulates activity and enantioselectivity in epoxide ring opening

Marcel Staar[a]+, Lina Ahlborn[a]+, Miquel Estévez-Gay[b], Katharina Pallasch[a], Sílvia Osuna*[b,c], Anett Schallmey*[a,d,e]

[a] M. Staar, L. Ahlborn, K. Pallasch, Prof. A. Schallmey
Institute for Biochemistry, Biotechnology and Bioinformatics, Technische Universität Braunschweig, Spielmannstr.7, 38106 Braunschweig (Germany)

[b] Dr. Miquel Estévez-Gay, Prof. Dr. Sílvia Osuna
CompBioLab Group, Institut de Química Computacional i Catàlisi (IQCC), Departament de Química, Universitat de Girona, c/Maria Aurèlia Capmany 69, 17003 Girona, Catalonia, Spain

[c] Prof. Dr. Sílvia Osuna
ICREA, Passeig Lluís Companys 23, 08010 Barcelona, Catalonia, Spain

[d] Prof. A. Schallmey
Zentrum für Pharmaverfahrenstechnik (PVZ), Technische Universität Braunschweig, Franz-Liszt-Str. 35a, 38106 Braunschweig (Germany)

[e] Prof. A. Schallmey
Braunschweig Integrated Center of Systems Biology (BRICS), Technische Universität Braunschweig, Rebenring 56, 38106 Braunschweig (Germany)

*Correspondence:
Prof. Dr. Anett Schallmey, Institute for Biochemistry, Biotechnology and Bioinformatics, Technical University Braunschweig, Spielmannstr. 7, 38106 Braunschweig, Germany.
E-mail: a.schallmey@tu-braunschweig.de

Prof. Dr. Sílvia Osuna, Institut de Química Computacional i Catàlisi (IQCC), Departament de Química, Universitat de Girona, c/Maria Aurèlia Capmany 69, 17003 Girona, Catalonia, Spain
E-mail: silviaosu@gmail.com

+ Both authors contributed equally to this work

Content

Author contributions 3
HheG homologs 4
Enzyme production in MTP format 5
Characterization data for all generated HheG variants 6
Computational analysis of HheG wild type and variant M45F 16
CLEC generation and characterization 19
Materials and Methods 20
Chemicals 20
Bacterial strains and plasmids 20
Mutagenesis 20
Protein production in 100 mL scale and purification via gravity-flow 22
Protein production in 500 mL scale and purification via FPLC 22
Protein crystallization and cross-linking 22
Biocatalysis using purified enzymes 23
Preparative-scale reaction 23
Thermal shift assay 23
Halide release assay 23
Gas chromatography (GC) 24
References 25

Author contributions

Conceptualization, A.S. and M.S.; Data curation, M.S., L.A. and M.E.; Formal analysis, M.S., L.A. and M.E.; Funding acquisition, A.S. and S.O.; Investigation, M.S., L.A., M.E. and K.P.; Methodology, M.S., L.A. and M.E.; Project administration, A.S.; Resources, A.S. and S.O.; Software, M.E. and S.O.; Supervision, A.S. and S.O.; Validation, - ; Visualization, M.S. and M.E.; Writing—original draft, M.S., A.S., M.E. and S.O.; Writing—review & editing, all authors.

Specifically regarding the experiments, M.S. and L.A. generated all mutants on the genetic level, produced and purified them, and performed all wet-lab enzyme characterizations assisted by K.P. Moreover, M.S. carried out the CLEC generation and characterization as well as their application in a semi-preparative reaction. M.E. performed all computational analyses including QM and MD simulations.

HheG homologs

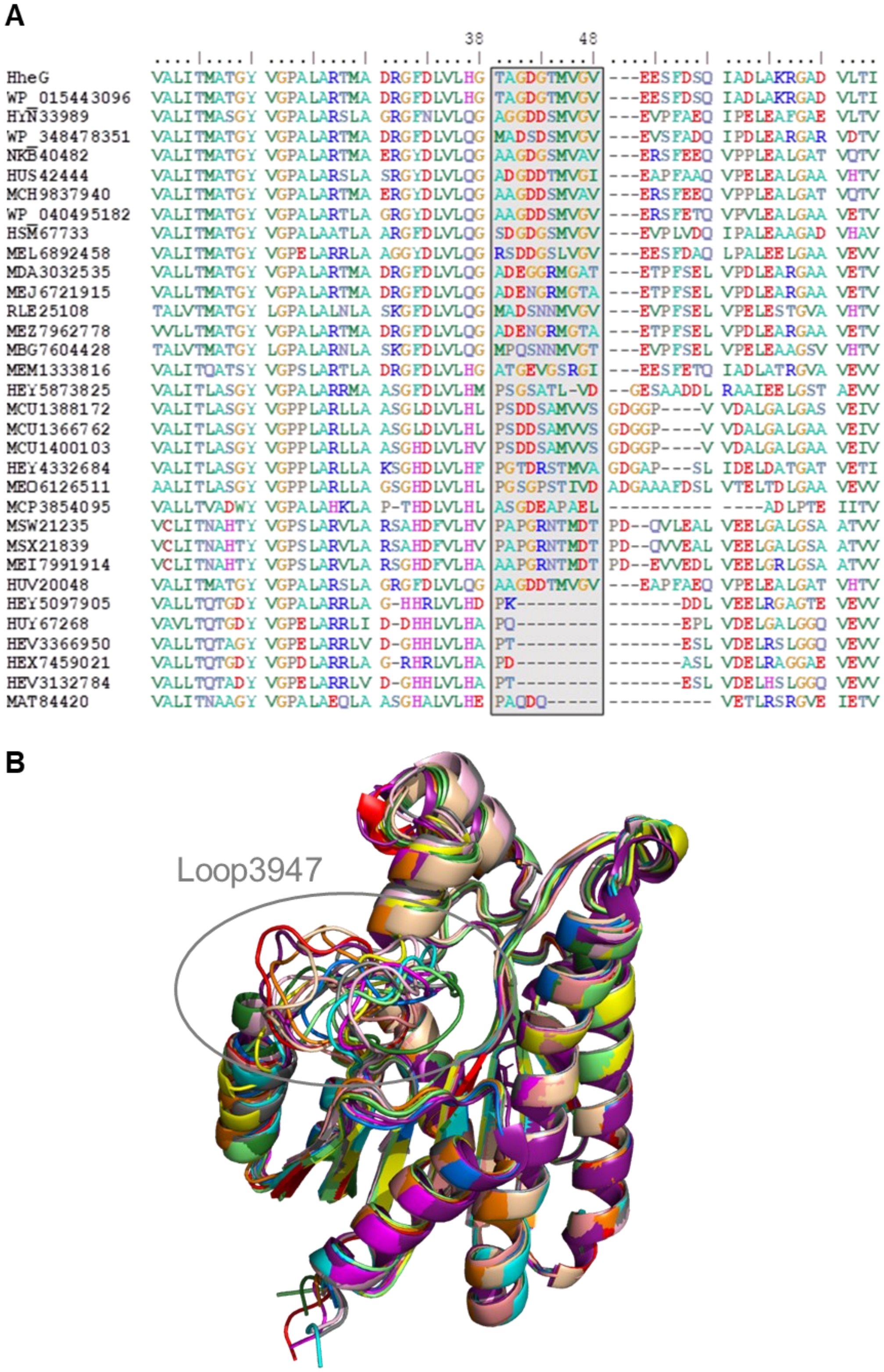

Figure S1. A. Excerpt of a multiple sequence alignment (generated by ClustalOmega) of HheG and 32 homologs, exhibiting 50 to 78% sequence identity on protein level with HheG, identified by BLAST search in the nr database of GenBank (release 262). Protein sequences are labelled according to their GenBank accession number. Residues corresponding to loop3947 of HheG are highlighted by a black frame and grey background. 24 G-type homologs feature a loop like HheG. **B.** Structural overlay of HheG (crystal structure, PDB: 5o30) and homology models of 12 selected G-type homologs (generated via AlphaFold3) featuring a similar flexible N-terminal loop as HheG [coloring scheme: HheG shown in

deep purple, HEY4332684 in lime, HEY5873825 in cyan, HYN33989 in magenta, MCU1388172 in yellow, MDA3032535 in salmon, MEI7991914 in grey, MEL6892458 in marine, MEM1333816 in orange, MEO6126511 in forest green, MSW21235 in light pink, RLE25108 in red and WP_348478351 in wheat]. For better visibility, only one monomer of the homotetrameric structure is shown in each case.

Enzyme production in MTP format

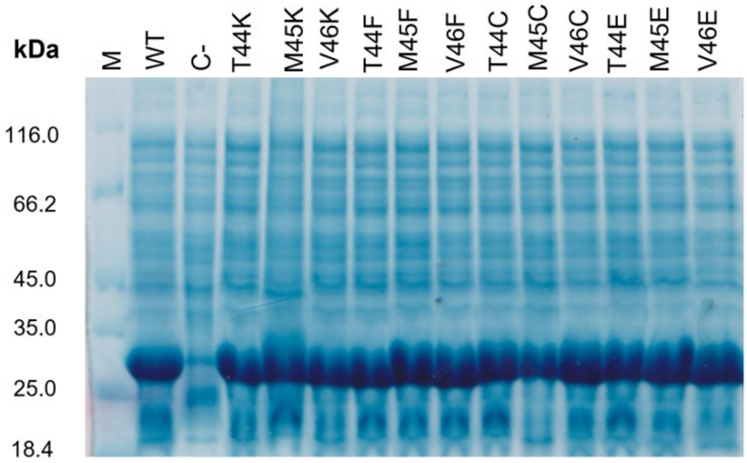

Figure S2. SDS-PAGE analysis example of protein production of the reduced loop3947 library of HheG in *E. coli* BL21(DE3) in 96-deep well plates. Variants with amino acid exchanges at positions T44, M45 and V46 are displayed. Wild-type HheG carrying an N-terminal hexahistidine-tag exhibits a molecular weight of 29.9 kDa. Pierce Unstained Protein MW Marker (ThermoFisher Scientific) was used as protein standard (M). “C-“ indicates the negative control of *E. coli* BL21(DE3) containing empty pET28a(+).

Characterization data for all generated HheG variants

Table S1. Characterization data (C: conversion, ee_P: product enantiomeric excess) of HheG variants of the fully randomized library at positions T44, M45 and V46 in the azidolysis of cyclohexene oxide (**1**). Reactions were performed at 22 °C and 900 rpm in 1 mL 50 mM Tris·SO_4 buffer, pH 7.0 using 200 µL CFE, 20 mM cyclohexene oxide (**1**) and 40 mM sodium azide. Samples were taken after 2 h and analyzed by achiral and chiral GC. ee_P is given for product enantiomer (1*S*,2*S*)-2-azidocyclohexan-1-ol (**2a**).

Position / Incorporated residue	T44		M45		V46	
	C [%]	ee_P [%]	C [%]	ee_P [%]	C [%]	ee_P [%]
G	95	65	94	69	92	68
A	95	65	94	66	94	68
V	95	56	93	68	97	49
L	95	58	95	76	93	68
I	94	61	95	68	93	63
M	94	63	97	49	93	67
P	94	66	93	59	94	69
W	95	64	95	92	95	69
F	61	46	93	96	28	56
Y	92	51	95	86	91	64
T	97	49	94	69	91	65
S	95	46	93	67	94	68
Q	95	67	93	68	89	68
N	95	66	92	68	95	69
C	92	56	29	61	94	70
R	87	68	85	68	97	67
K	55	67	97	69	94	68
H	95	71	94	73	82	69
D	95	63	94	69	94	69
E	93	70	85	68	91	69

Table S2. Characterization data (C: conversion, ee_P: product enantiomeric excess) of HheG variants of the fully randomized library at positions T44, M45 and V46 in the cyanolysis of cyclohexene oxide (**1**). Reactions were performed at 22 °C and 900 rpm in 1 mL 50 mM Tris·SO_4 buffer, pH 7.0 using 200 µL CFE, 20 mM cyclohexene oxide (**1**) and 40 mM sodium cyanide. Samples were taken after 24 h and analyzed by achiral and chiral GC. ee_P is given for product enantiomer (1*R*,2*S*)-2-cyanocyclohexan-1-ol (**2b**).

Position / Incorporated residue	T44		M45		V46	
	C [%]	ee_P [%]	C [%]	ee_P [%]	C [%]	ee_P [%]
G	11	13	11	0.5	11	1.9
A	29	4.8	12	3.3	11	-0.8
V	17	20	12	1.7	22	29
L	22	22	17	-10	11	-1.0
I	13	11	14	2.4	13	7.4
M	18	15	22	29	11	1.4
P	12	3.6	11	7.0	26	0.9
W	21	17	17	-28	17	1.8
F	42	40	66	-60	12	8.6
Y	36	35	16	-20	12	5.7
T	22	29	13	-0.1	13	3.6
S	40	21	11	1.7	11	1.4
Q	23	9.5	11	0.4	26	0.6
N	20	8.2	12	2.7	12	-0.4
C	34	14	7.8	4.5	17	-8.3
R	7.8	2.1	7.4	0.3	5.4	1.1
K	8.7	2.2	8.4	0.2	8.5	0.2
H	21	3.7	9.6	-3.2	9.0	0.7
D	16	1.9	13	0.8	13	0.3
E	20	4.9	14	0.0	13	-0.5

Table S3. Characterization data (C: conversion, ee_P: product enantiomeric excess, E: enantioselectivity) of HheG variants of the fully randomized library at positions T44, M45 and V46 in the azidolysis of styrene oxide (**3**). Reactions were performed at 22 °C and 900 rpm in 1 mL 50 mM Tris·SO_4 buffer, pH 7.0 using 100 µL CFE, 20 mM styrene oxide (**3**) and 40 mM sodium azide. Samples were taken after 10 min and analyzed by chiral GC. ee_P is given for product enantiomer (*S*)-2-azidophenylethan-1-ol (**4**).

Position / Incorporated residue	T44			M45			V46		
	C [%]	ee_P [%]	E	C [%]	ee_P [%]	E	C [%]	ee_P [%]	E
G	59	67	20	57	72	24	58	71	25
A	57	71	22	60	65	19	59	67	21
V	56	77	30	62	60	17	59	69	23
L	56	77	31	57	72	22	55	78	28
I	53	81	29	59	68	20	59	68	21
M	55	77	30	59	69	23	58	70	22
P	62	61	16	60	65	20	58	71	24
W	55	79	32	55	78	28	57	71	22
F	55	80	40	53	84	46	55	74	22
Y	55	79	36	55	78	31	57	73	26
T	59	69	23	58	69	21	58	70	23
S	59	68	22	61	63	17	58	72	25
Q	58	71	24	59	68	21	57	74	25
N	59	68	21	59	69	22	58	72	26
C	57	72	25	59	66	20	58	70	23
R	54	79	27	54	79	26	48	82	23
K	53	79	24	52	79	23	53	79	26
H	59	68	22	56	76	29	55	77	27
D	61	63	18	62	60	16	59	67	21
E	61	62	17	59	67	21	58	69	22

Table S4. Conversion (C) and regioisomeric ratio of products 2-azidoheptan-3-ol (**6a**) and 3-azidoheptan-2-ol (**6b**) of HheG variants of the fully randomized library at positions T44, M45 and V46 in the azidolysis of *trans*-2,3-heptene oxide (**5**). Reactions were performed at 22 °C and 900 rpm in 1 mL 50 mM Tris·SO_4 buffer, pH 7.0 using 200 µL CFE, 10 mM *trans*-2,3-heptene oxide (**5**) and 20 mM sodium azide. Samples were taken after 30 min and analyzed by achiral GC.

**Position** / **Incorporated residue**	**T44**		**M45**		**V46**	
	**C [%]**	Regioisomeric ratio **6a**:**6b**	**C [%]**	Regioisomeric ratio **6a**:**6b**	**C [%]**	Regioisomeric ratio **6a**:**6b**
G	82	48:52	69	47:53	62	46:54
A	84	47:53	67	45:55	71	47:53
V	92	48:52	73	47:53	100	48:52
L	93	50:50	43	39:61	49	45:55
I	83	48:52	55	43:57	72	45:55
M	89	49:51	100	48:52	62	46:54
P	81	48:52	46	44:56	65	48:52
W	88	50:50	57	50:50	51	40:60
F	100	50:50	85	49:51	64	41:59
Y	99	50:50	59	53:47	54	45:55
T	100	48:52	71	47:53	60	46:54
S	97	48:52	65	43:57	68	47:53
Q	88	48:52	68	47:53	46	45:55
N	89	49:51	60	46:54	67	47:53
C	86	48:52	68	45:55	84	47:53
R	55	46:54	42	45:55	23	44:56
K	52	44:56	54	44:56	55	46:54
H	82	49:51	58	46:54	45	45:55
D	76	47:53	77	47:53	74	48:52
E	82	47:53	68	47:53	71	48:52

Table S5. Conversion (C), product enantiomeric excess (ee_P) and E value for product regioisomer 2-azidoheptan-3-ol (**6a**) of HheG variants of the fully randomized library at positions T44, M45 and V46 in the azidolysis of *trans*-2,3-heptene oxide (**5**). Reactions were performed at 22 °C and 900 rpm in 1 mL 50 mM Tris·SO_4 buffer, pH 7.0 using 200 µL CFE, 10 mM *trans*-2,3-heptene oxide (**5**) and 20 mM sodium azide. Samples were taken after 30 min and analyzed by achiral and chiral GC.

Position / Incorporated residue	T44			M45			V46		
	C [%]	ee_P [%]	E	C [%]	ee_P [%]	E	C [%]	ee_P [%]	E
G	39	1.3	1.0 (2*R*,3*S*)	33	5.3	1.1 (2*R*,3*S*)	28	13	1.4 (2*R*,3*S*)
A	40	1.7	1.0 (2*S*,3*R*)	30	5.3	1.1 (2*R*,3*S*)	34	3.1	1.1 (2*R*,3*S*)
V	44	9.2	1.3 (2*S*,3*R*)	34	2.9	1.1 (2*S*,3*R*)	48	12	1.4 (2*S*,3*R*)
L	47	8.8	1.3 (2*S*,3*R*)	17	35	2.2 (2*R*,3*S*)	22	24	1.7 (2*R*,3*S*)
I	39	2.4	1.1 (2*S*,3*R*)	24	18	1.5 (2*R*,3*S*)	32	2.0	1.0 (2*R*,3*S*)
M	44	6.5	1.2 (2*S*,3*R*)	48	12	1.4 (2*S*,3*R*)	29	7.0	1.2 (2*R*,3*S*)
P	38	2.1	1.1 (2*S*,3*R*)	20	27	1.9 (2*R*,3*S*)	31	8.8	1.2 (2*R*,3*S*)
W	44	3.7	1.1 (2*S*,3*R*)	28	35	2.4 (2*R*,3*S*)	20	25	1.8 (2*R*,3*S*)
F	50	14	1.5 (2*S*,3*R*)	41	3.6	1.1 (2*R*,3*S*)	26	14	1.4 (2*R*,3*S*)
Y	49	13	1.4 (2*S*,3*R*)	31	26	1.9 (2*R*,3*S*)	24	19	1.6 (2*R*,3*S*)
T	48	12	1.4 (2*S*,3*R*)	33	0.7	1.0 (2*S*,3*R*)	28	12	1.3 (2*R*,3*S*)
S	47	8.3	1.3 (2*S*,3*R*)	28	4.2	1.1 (2*R*,3*S*)	32	8.0	1.2 (2*R*,3*S*)
Q	43	4.8	1.1 (2*S*,3*R*)	32	5.8	1.2 (2*R*,3*S*)	21	28	1.9 (2*R*,3*S*)
N	43	5.9	1.2 (2*S*,3*R*)	28	13	1.4 (2*R*,3*S*)	32	7.1	1.2 (2*R*,3*S*)
C	41	4.2	1.1 (2*S*,3*R*)	31	4.7	1.1 (2*R*,3*S*)	40	5.9	1.2 (2*S*,3*R*)
R	25	20	1.6 (2*R*,3*S*)	19	28	1.9 (2*R*,3*S*)	10	46	2.8 (2*R*,3*S*)
K	23	34	2.2 (2*R*,3*S*)	24	33	2.2 (2*R*,3*S*)	26	17	1.5 (2*R*,3*S*)
H	40	0.9	1.0 (2*S*,3*R*)	27	21	1.7 (2*R*,3*S*)	20	28	1.9 (2*R*,3*S*)
D	35	0.8	1.0 (2*R*,3*S*)	37	8.6	1.2 (2*S*,3*R*)	35	1.2	1.0 (2*S*,3*R*)
E	38	0.6	1.0 (2*S*,3*R*)	32	3.7	1.1 (2*R*,3*S*)	34	3.0	1.1 (2*R*,3*S*)

Table S6. Conversion (C), product enantiomeric excess (ee_P) and E value for product regioisomer 3-azidoheptan-2-ol (**6b**) of HheG variants of the fully randomized library at positions T44, M45 and V46 in the azidolysis of *trans*-2,3-heptene oxide (**5**). Reactions were performed at 22 °C and 900 rpm in 1 mL 50 mM Tris·SO_4 buffer, pH 7.0 using 200 µL CFE, 10 mM *trans*-2,3-heptene oxide (**5**) and 20 mM sodium azide. Samples were taken after 30 min and analyzed by achiral and chiral GC. ee_P is given for product enantiomer (2*S*,3*R*)-3-azidoheptan-2-ol (**6b**).

Position / Incorporated residue	T44			M45			V46		
	C [%]	ee_P [%]	E	C [%]	ee_P [%]	E	C [%]	ee_P [%]	E
G	43	29	2.2	36	40	2.9	33	42	3.0
A	44	27	2.1	37	45	3.3	38	37	2.7
V	48	17	1.6	38	39	2.9	52	12	1.4
L	47	18	1.7	26	66	6.1	27	57	4.5
I	43	24	2.0	31	53	4.1	40	38	2.8
M	45	21	1.8	52	12	1.4	33	45	3.3
P	42	29	2.2	26	58	4.5	34	46	3.3
W	44	23	1.9	28	37	2.5	30	62	5.4
F	50	15	1.5	44	17	1.6	38	56	4.9
Y	50	16	1.6	28	41	2.8	29	52	3.9
T	52	12	1.4	38	40	3.0	32	47	3.5
S	50	16	1.6	37	46	3.5	37	42	3.0
Q	46	23	1.9	36	43	3.2	25	59	4.7
N	46	22	1.9	33	50	3.7	35	44	3.2
C	44	23	1.9	37	45	3.4	44	27	2.1
R	29	50	3.7	23	59	4.6	13	70	6.3
K	29	60	5.1	30	62	5.5	30	50	3.7
H	42	27	2.1	32	52	4.0	25	60	4.8
D	40	33	2.4	41	36	2.7	38	38	2.7
E	44	32	2.4	36	40	2.9	37	41	3.0

Table S7. Specific activities of selected purified HheG variants in indicated epoxide ring opening reactions as determined by BTB assay, as well as apparent melting temperatures (T_m) of respective variants analyzed by thermofluor assay. Specific activities were determined in duplicate. T_m values were obtained from triplicate measurements.

**Substrate**	**Variant**	**Specific activity [U mg^{-1}]**	**T_m [°C]**
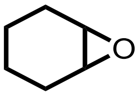 **N_3^-**	WT	1.2 ± 0.0	40.0 ± 0.2
	M45F	11 ± 1.2	41.2 ± 0.2
	M45Y	3.5 ± 0.4	39.2 ± 0.3
	M45W	0.8 ± 0.0	39.3 ± 0.1
	M45F-T123G	6.7 ± 0.2	48.0 ± 0.1
	M45F-T123F	5.1 ± 0.4	48.0 ± 0.2
	M45Y-T123G	3.0 ± 0.4	48.0 ± 0.1
	M45Y-T123F	2.1 ± 0.0	52.0 ± 0.0
	M45W-T123G	4.2 ± 0.4	52.0 ± 0.1
	M45W-T123F	1.1 ± 0.0	52.0 ± 0.1
	Del44-46	0.3 ± 0.0	38.2 ± 0.1
	Ins-DPAE	0.2 ± 0.0	39.4 ± 0.2
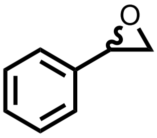 **N_3^-**	WT	2.4 ± 0.4	
	T44F	8.5 ± 0.4	41.2 ± 0.2
	T44Y	3.0 ± 0.0	39.2 ± 0.3
	T44W	1.6 ± 0.0	39.3 ± 0.1
	M45F	5.3 ± 0.6	
	M45F-T123G	15 ± 0.0	
	M45F-T123F	4.7 ± 0.3	
	M45Y-T123G	10 ± 0.4	
	M45Y-T123F	6.7 ± 0.0	
	M45W-T123G	9.2 ± 0.3	
	M45W-T123F	1.0 ± 0.0	
	Del44-46	9.9 ± 2.9	
	Ins-DPAE	7.5 ± 2.4	
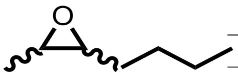 **N_3^-**	WT	0.7 ± 0.1	
	T44K	0.1 ± 0.0	41.2 ± 0.2
	M45L	0.2 ± 0.0	39.2 ± 0.3
	M45K	0.04 ± 0.00	39.3 ± 0.1
	V46R	n.d.[a]	48.0 ± 0.1
	M45F-T123G	0.6 ± 0.1	
	M45F-T123F	0.5 ± 0.1	
	M45Y-T123G	0.2 ± 0.0	
	M45Y-T123F	0.1 ± 0.0	
	M45W-T123G	0.4 ± 0.0	
	M45W-T123F	0.2 ± 0.1	
	Del44-46	n.d.[a]	
	Ins-DPAE	n.d.[a]	

[a] not detectable

Table S8. Characterization data of purified HheG variants in indicated epoxide ring opening reactions using different nucleophiles. Reactions were performed at 22 °C in 50 mM Tris·SO_4 buffer, pH 7.0 for the indicated amount of time and analyzed by achiral and chiral GC. All reactions were performed in duplicate.

Substrate	Variant	Conversion [%]		Product enantiomeric excess [%]		E value	
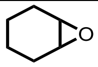 N_3^-	WT	71 ± 0.2[a]		49 ± 0.4 (*1S,2S*)[a]		-	
	M45F-T123G	81 ± 1.1[a]		88 ± 0.0 (*1S,2S*)[a]		-	
	M45F-T123F	79 ± 1.1[a]		92 ± 0.6 (*1S,2S*)[a]		-	
	M45Y-T123G	77 ± 0.2[a]		67 ± 0.4 (*1S,2S*)[a]		-	
	M45Y-T123F	83 ± 7.2[a]		79 ± 0.7 (*1S,2S*)[a]		-	
	M45W-T123G	88 ± 6.5[a]		93 ± 0.2 (*1S,2S*)[a]		-	
	M45W-T123F	79 ± 1.4[a]		95 ± 0.5 (*1S,2S*)[a]		-	
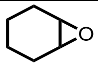 CN^-	WT	20 ± 0.5[b]		28 ± 0.0 (*1R,2S*)[b]		-	
	M45F-T123G	93 ± 2.2[b]		41 ± 0.2 (*1S,2R*)[b]		-	
	M45F-T123F	73 ± 0.1[b]		46 ± 0.1 (*1S,2R*)[b]		-	
	M45Y-T123G	71 ± 3.2[b]		7 ± 0.2 (*1R,2S*)[b]		-	
	M45Y-T123F	37 ± 5.9[b]		8 ± 0.4 (*1S,2R*)[b]		-	
	M45W-T123G	78 ± 3.8[b]		61 ± 0.2 (*1S,2R*)[b]		-	
	M45W-T123F	40 ± 2.5[b]		50 ± 0.2 (*1S,2R*)[b]		-	
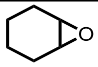 OCN^-	WT	54 ± 0.9[b]		57 ± 0.3[b,e]		-	
	M45F-T123G	99 ± 0.0[b]		9.6 ± 0.1[b,e]		-	
	M45F-T123F	93 ± 1.8[b]		21 ± 0.1[b,e]		-	
	M45Y-T123G	86 ± 0.1[b]		-38 ± 0.6[b,e]		-	
	M45Y-T123F	46 ± 2.2[b]		-18 ± 0.0[b,e]		-	
	M45W-T123G	98 ± 1.1[b]		35 ± 0.3[b,e]		-	
	M45W-T123F	53 ± 3.6[b]		38 ± 0.0[b,e]		-	
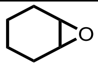 NO_2^-	WT	15 ± 0.1[b] (73:27)[f]		0.1 ± 0.1[b,e]		-	
	M45F-T123G	100 ± 0.0[b] (66:34)[f]		31 ± 0.0[b,e]		-	
	M45F-T123F	99± 0.0[b] (62:38)[f]		37 ± 0.1[b,e]		-	
	M45Y-T123G	94 ± 0.1[b] (73:27)[f]		-4.2 ± 0.1[b,e]		-	
	M45Y-T123F	40 ± 0.3[b] (71:29)[f]		13 ± 0.1[b,e]		-	
	M45W-T123G	100 ± 0.2[b] (52:48)[f]		42 ± 0.3[b,e]		-	
	M45W-T123F	44 ± 0.1[b] (50:50)[f]		34 ± 0.7[b,e]		-	
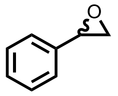 N_3^-	WT	46 ± 1.4[c]		84 ± 0.4 (*2S*)[c]		24 ± 1.9	
	M45F-T123G	48 ± 0.3[c]		85 ± 0.0 (*2S*)[c]		29 ± 0.4	
	M45F-T123F	42 ± 2.2[c]		86 ± 0.0 (*2S*)[c]		25 ± 1.8	
	M45Y-T123G	47 ± 1.1[c]		88 ± 0.3 (*2S*)[c]		37 ± 1.0	
	M45Y-T123F	52 ± 0.7[c]		85 ± 0.5 (*2S*)[c]		38 ± 1.5	
	M45W-T123G	46 ± 1.0[c]		87 ± 0.1 (*2S*)[c]		32 ± 1.2	
	M45W-T123F	46 ± 0.3[c]		84 ± 0.3 (*2S*)[c]		24 ± 0.9	
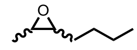 N_3^-		**6a**	**6b**	**6a**	**6b**	**6a**	**6b**
	WT	48 ± 0.1[d]	51 ± 0.1[d]	12 ± 0.3 (*2S,3R*)[d]	12 ± 0.0 (*2S,3R*)[d]	1.4 ± 0.0	1.4 ± 0.0
	M45F-T123G	41 ± 1.0[d]	49 ± 1.3[d]	10 ± 3.2 (*2R,3S*)[d]	6.9 ± 2.8 (*2S,3R*)[d]	1.3 ± 0.1	1.2 ± 0.1
	M45F-T123F	45 ± 0.6[d]	47 ± 0.5[d]	0.1 ± 0.1 (*2S,3R*)[d]	14 ± 1.7 (*2S,3R*)[d]	1.0 ± 0.0	1.5 ± 0.0
	M45Y-T123G	48 ± 0.6[d]	37 ± 0.4[d]	7.7 ± 1.8 (*2R,3S*)[d]	14 ± 0.6 (*2S,3R*)[d]	1.2 ± 0.0	1.4 ± 0.0
	M45Y-T123F	39 ± 0.4[d]	33 ± 0.3[d]	13 ± 1.7 (*2R,3S*)[d]	38 ± 1.3 (*2S,3R*)[d]	1.4 ± 0.1	2.6 ± 0.1
	M45W-T123G	40 ± 0.8[d]	42 ± 0.9[d]	29 ± 3.1 (*2R,3S*)[d]	1.9 ± 2.9 (*2S,3R*)[d]	2.2 ± 0.2	1.1 ± 0.1
	M45W-T123F	38 ± 0.0[d]	36 ± 0.2[d]	23 ± 0.0 (*2R,3S*)[d]	25 ± 0.1 (*2S,3R*)[d]	1.8 ± 0.0	1.9 ± 0.0

[a] determined after 2 h
[b] determined after 24 h
[c] determined after 10 min
[d] determined after 3 h
[e] enantiomers unassigned
[f] product ratio of nitroalcohol **2c**:diol **2f**

Table S9. Characterization data of purified HheG deletion variants in indicated epoxide ring opening reactions using different nucleophiles. Reactions were performed at 22 °C in 50 mM Tris·SO_4 buffer, pH 7.0 for the indicated amount of time and analyzed by achiral and chiral GC. All reactions were performed in duplicate except for reactions of HheG variants Del44-46 and Ins-DPAE with *trans*-2,3-heptene oxide (**5**), which were performed only once.

Substrate	Variant	Conversion [%]		Product enantiomeric excess [%]		E value	
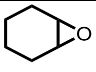	WT	71 ± 0.2[a]		49 ± 0.4 (*1S,2S*)[a]		-	
	Del44-46	77 ± 0.2[a]		77 ± 8.7 (*1S,2S*)[a]		-	
**N_3^-**	Ins-DPAE	70 ± 0.2[a]		57 ± 0.4 (*1S,2S*)[a]		-	
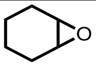	WT	20 ± 0.5[b]		28 ± 0.0 (*1R,2S*)[b]		-	
	Del44-46	13 ± 0.1[b]		1.4 ± 0.3 (*1S,2R*)[b]		-	
**CN^-**	Ins-DPAE	12 ± 0.0[b]		0.5 ± 0.4 (*1S,2R*)[b]		-	
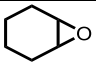	WT	54 ± 0.9[b]		57 ± 0.3[b,e]		-	
	Del44-46	19 ± 0.1[b]		32 ± 0.0[b,e]		-	
**OCN^-**	Ins-DPAE	16 ± 0.1[b]		36 ± 0.1[b,e]		-	
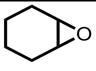	WT	15 ± 0.1[b] (73:27)[f]		0.1 ± 0.1[b,e]		-	
	Del44-46	11 ± 0.4[b] (72:28)[f]		6.2 ± 0.3[b,e]		-	
**NO_2^-**	Ins-DPAE	11 ± 0.1[b] (76:24)[f]		4.0 ± 0.3[b,e]		-	
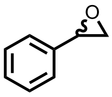	WT	46 ± 1.4[c]		84 ± 0.4 (*2S*)[c]		24 ± 1.9	
	Del44-46	32 ± 0.2[c]		84 ± 0.1 (*2S*)[c]		16 ± 0.1	
**N_3^-**	Ins-DPAE	31 ± 0.1[c]		83 ± 0.25 (*2S*)[c]		16 ± 0.2	
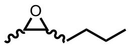		**6a**	**6b**	**6a**	**6b**	**6a**	**6b**
**N_3^-**	WT	48 ± 0.1[d]	51 ± 0.1[d]	12 ± 0.3 (*2S,3R*)[d]	12 ± 0.0 (*2S,3R*)[d]	1.4 ± 0.0	1.4 ± 0.0
	Del44-46	39[d]	44[d]	3.3 (*2S,3R*)[d]	36 (*2S,3R*)[d]	1.1	2.8
	Ins-DPAE	37[d]	41[d]	2.3 (*2R,3S*)[d]	43 (*2S,3R*)[d]	0.9	3.4

[a] determined after 2 h
[b] determined after 24 h
[c] determined after 10 min
[d] determined after 3 h
[e] enantiomers unassigned
[f] product ratio of nitroalcohol **2c**:diol **2f**

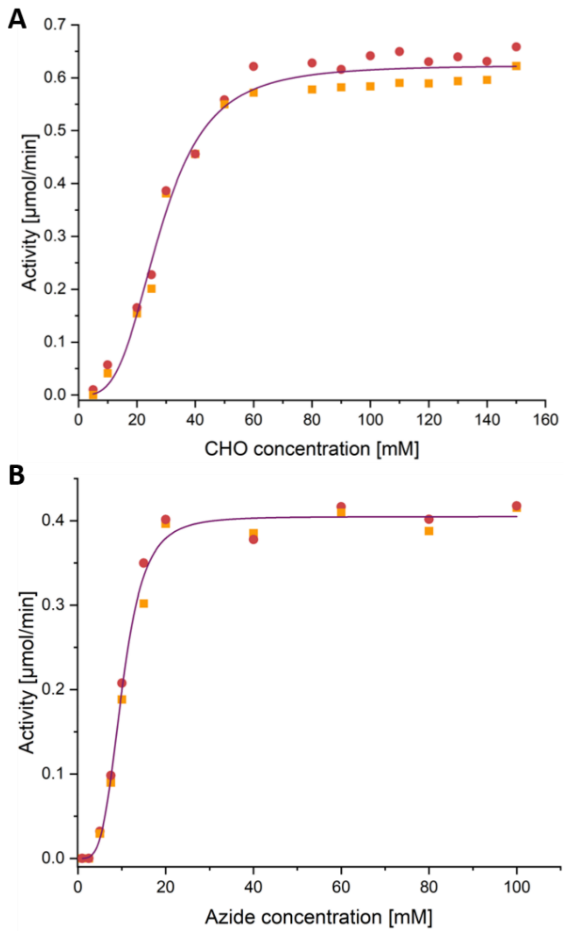

Figure S4. Kinetic measurements of HheG M45F in the ring opening of cyclohexene oxide (**1**, CHO) with azide as determined by BTB assay.[1] (**A**) The epoxide concentration was varied while the azide concentration was kept at 60 mM. (**B**) The azide concentration was varied while keeping the epoxide concentration constant at 100 mM. Reactions were performed in duplicate in 2 mM MOPS·SO_4 buffer, pH 7.0 at 22 °C. Samples were taken after 30, 60, 90 and 120s. Resulting data were fitted to the Hill equation for cooperative substrate binding in Origin Pro.

Computational analysis of HheG wild type and variant M45F

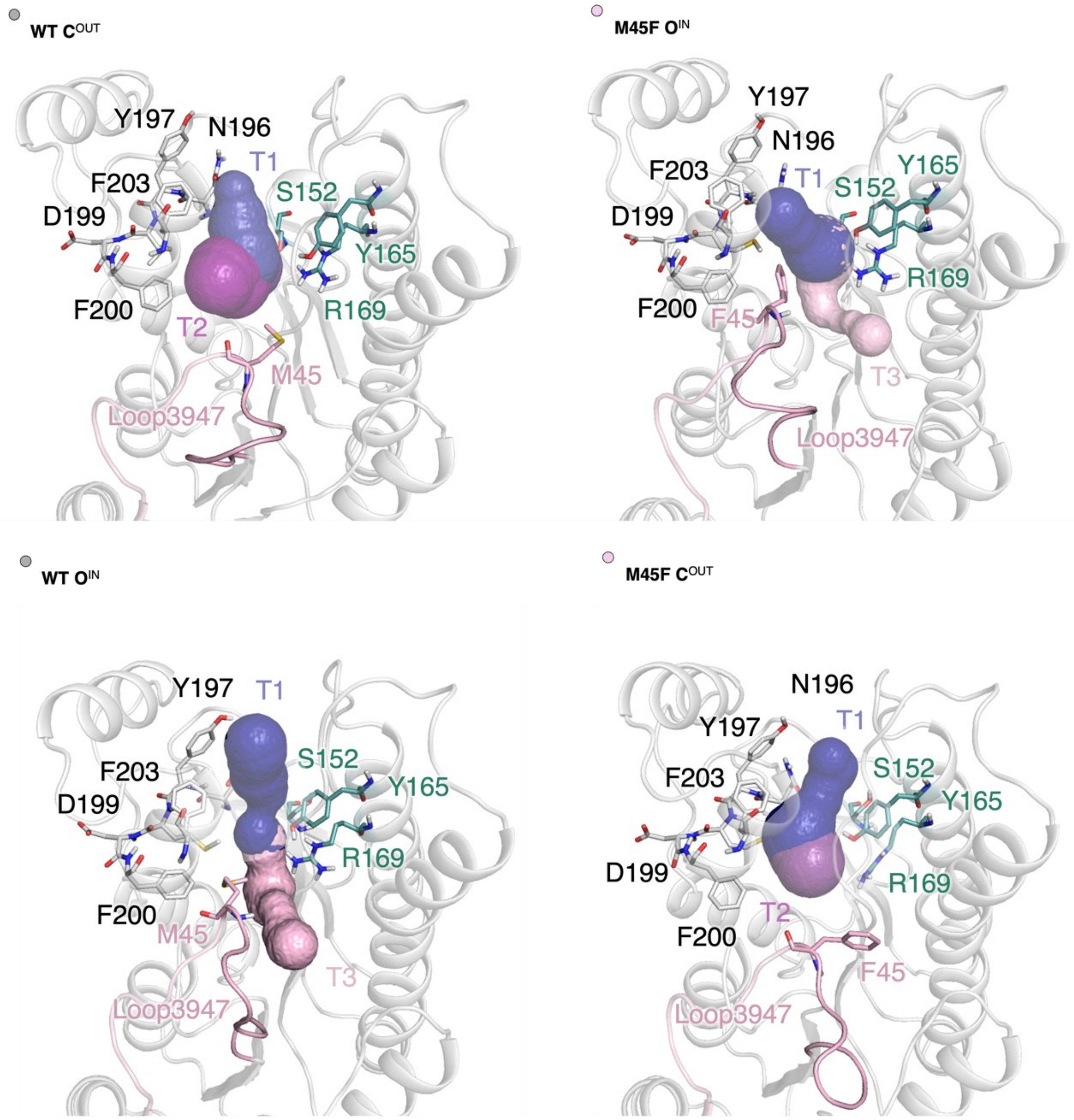

Figure S4. A representative structure of the most populated minima is displayed together with the available tunnels: **C**^{OUT} and **O**^{IN} conformation for HheG wild type (WT) and variant M45F. Nucleophile binding site residues are shown using gray sticks, catalytic residues in teal, and loop3947 and position 45 in light pink. Tunnel 1 (T1) is shown in dark blue, whereas tunnel 2 (T2) in purple and tunnel 3 (T3) in light pink.

Table S10. Tunnel 2 frequency and associated bottleneck radius for HheG wild type (WT) and variant M45F for the most stable conformation for each system (i.e. C^{OUT} and O^{IN} for HheG WT and M45F, respectively).

Variant	Tunnel 2 frequency	Bottleneck radius (in Å)
WT	92.5%	1.9±0.4 Å
M45F	71.8%	1.5±0.3 Å

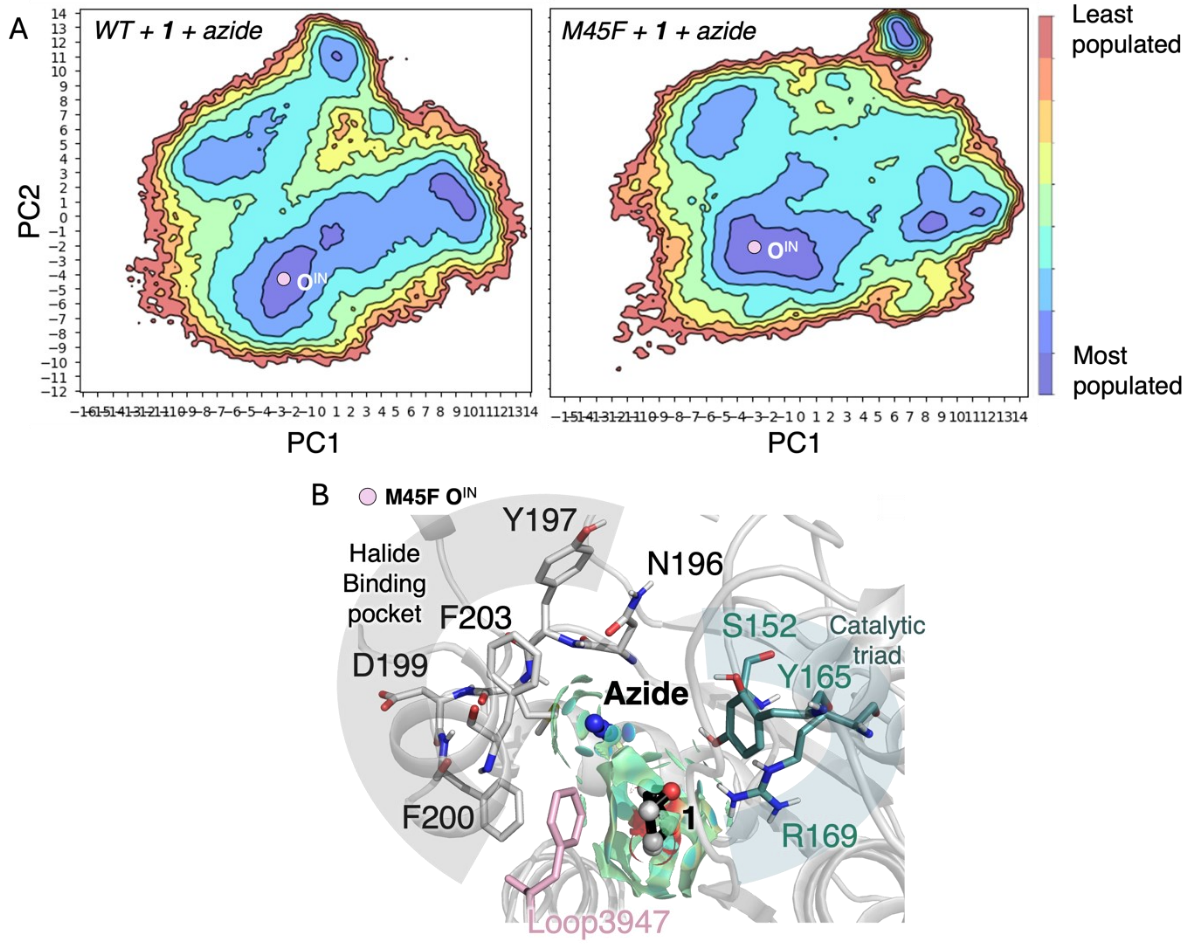

Figure S5. A. Estimated free energy landscapes (FEL) of HheG wild type (WT) and variant M45F in the presence of cyclohexene oxide (**1**) and azide. PC1 and PC2 describe the open/closed conformational change of loop3947 and the side chain orientation of M/F45 within the active site pocket. Most stable conformations are colored in blue, whereas least stable ones in red. **B.** Representation of the non-covalent interactions (as described by NCI analysis[2]) established between cyclohexene oxide (**1**) and the active site pocket including residue F45 in a representative protein structure of variant M45F taken from the substrate-bound MD simulations (taken from $\mathbf{O^{IN}}$ minima marked in FEL as a pink sphere). Strong attractive interactions are displayed with a blue mesh, weak non-covalent interactions in green, and repulsive interactions in red.

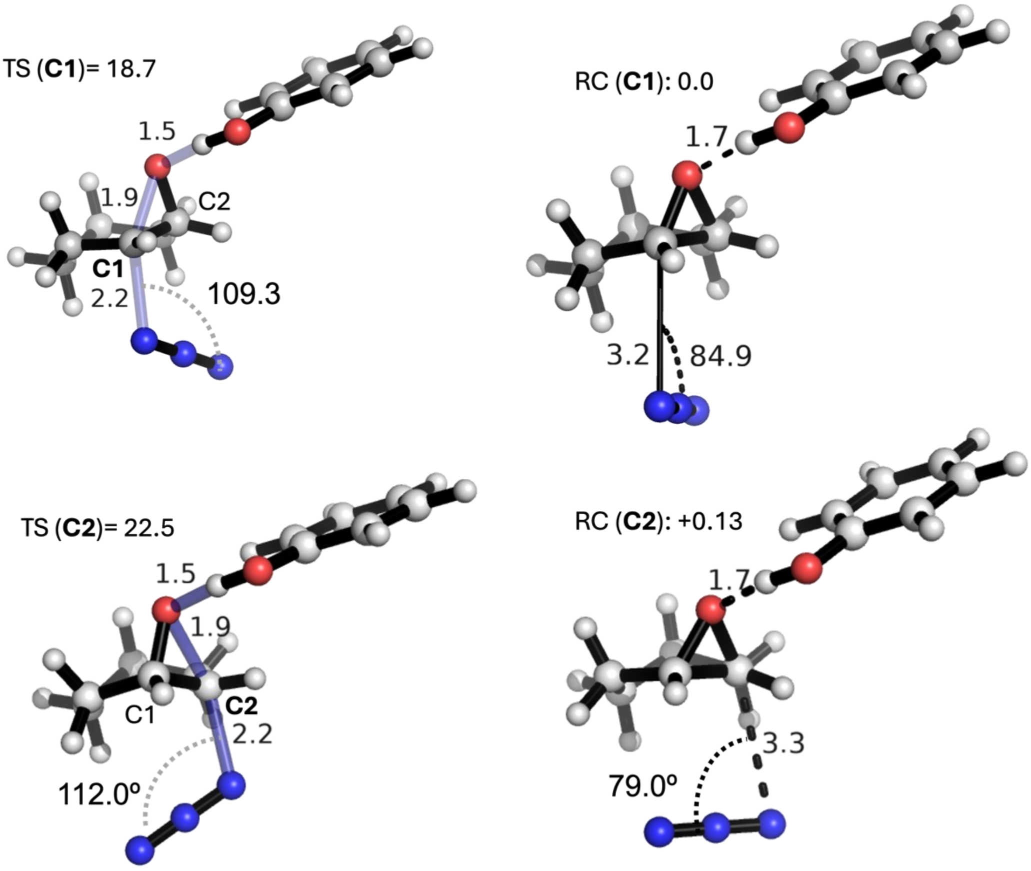

Figure S6. DFT optimized geometry and electronic energies (wB97XD/6-311+G(2d,2p)//B3LYP-D3/6-31+G(d,p)) of the transition state and the reactant complex of the cyclohexene oxide ring-opening reaction using azide as nucleophile with nucleophilic attack at C1 (leading to the *S*-enantiomer) and C2 (leading to the *R*-enantiomer). All distances are shown in Å, angles in degrees, and energies in kcal/mol.

Table S11. Estimation of the enantiomeric excess (ee) for HheG wild type (WT) and mutant M45F. We filtered the number of MD frames presenting catalytically competent poses by considering distances < 4 Å between the epoxide oxygen of **1** and the catalytic Tyr165, as well as the distance (< 4 Å) and angle (range of 80 to 120º) between azide and either C_1/C_2 of cyclohexene oxide corresponding to pro-*S*/pro-*R* attacks, respectively. $P_{R/S}$ is the productive number of pro-*R*/pro-*S* attacks computed considering the counting of productive pro-*R*/pro-*S* attacks ($c_{R/S}$) along the MD simulation divided by the total number of frames (N) in the MD simulation.

$$\%ee = \left(\frac{P_R - P_S}{P_R + P_S}\right) x100 \qquad P_R = \frac{C_R}{N} \qquad P_S = \frac{C_S}{N} \qquad \text{(eq. 1)}$$

System	WT	M45F
ee_{comp}	13.7%	40.7%

CLEC generation and characterization

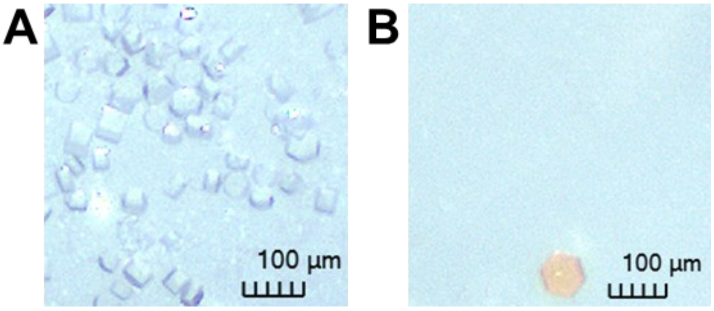

Figure S7. Hexagonal-shaped CLECs of HheG variant M45F-D114C after crystallization and cross-linking with bis-maleimidoethane for each 24 h (**A**) and after staining with SYPRO orange (**B**).

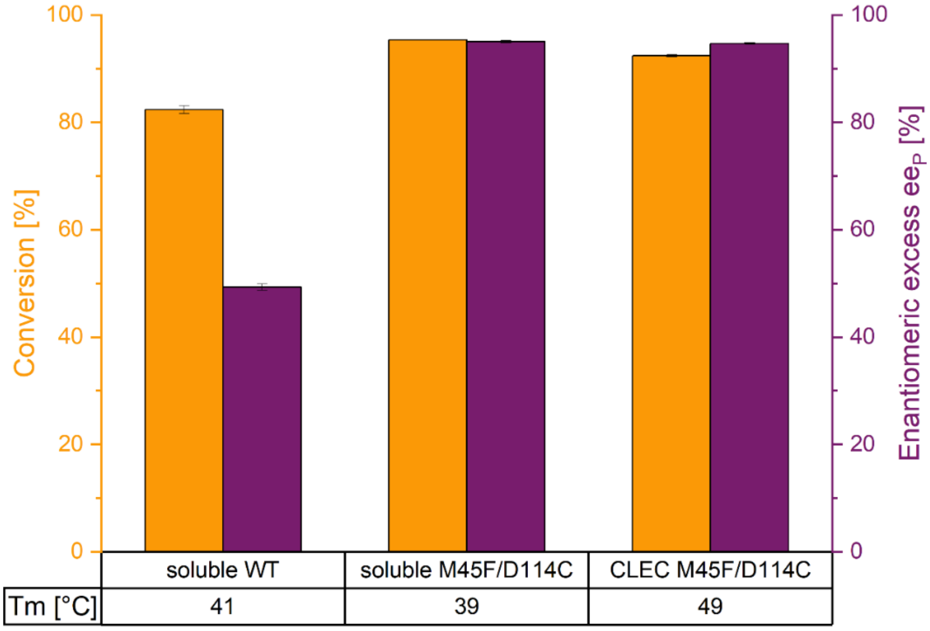

Figure S8. Characterization data (conversion, product enantiomeric excess and apparent melting temperature) of soluble HheG wild-type, soluble variant M45F-D114C and CLECs of HheG variant M45F-D114C in the azidolysis of cyclohexene oxide (**1**). Reactions were performed in 1 mL scale in 50 mM Tris·SO_4 buffer, pH 7.0 using 200 µg biocatalyst, 20 mM cyclohexene oxide (**1**) and 40 mM azide. Reactions were performed in duplicate and incubated for each 1 h at 22 °C and 900 rpm, and analyzed via achiral and chiral GC. Apparent melting temperatures (T_m) were determined in triplicate via thermal shift assay.

Materials and Methods

Chemicals

Substrates cyclohexene oxide (**1**) and styrene oxide (**3**) as well as crosslinker bis-maleimidoethane (BMOE) were purchased from Thermo Fisher Scientific (Geel, Belgium). Substrate *trans*-2,3-heptene-oxide (**5**) was synthesized according to Calderini et al.[3] starting from *trans*-2-heptene. Substrate 2-chlorocyclohexanol (**2e**) was purchased from TCI (Tokyo, Japan). All commercial chemicals were of highest available purity.

Bacterial strains and plasmids

E. coli BL21(DE3) Gold was used for cloning as well as heterologous protein production as described previously.[4] Further, expression vector pET-28a(+) (Merck) was used to carry respective mutant genes under control of the T7 promoter, resulting in the addition of an N-terminal His_6-tag to heterologously produced proteins.

Mutagenesis

Table S12. Mutagenic primers used in this study. Nucleotide exchanges are highlighted in bold.

Mutagenic primer	Sequence 5´-3´
f_hheG_T39K	GGTTCTGCATGGT**AAA**GCCGGTGATGGCAC
r_hheG_T39K	GTGCCATCACCGGC**TTT**ACCATGCAGAACC
f_hheG_T39E	CTGGTTCTGCATGGT**GAA**GCCGGTGATGGCAC
r_hheG_T39E	GTGCCATCACCGGC**TTC**ACCATGCAGAACCAG
f_hheG_T39F	GATCTGGTTCTGCATGGT**TTT**GCCGGTGATGGCACCATGG
r_hheG_T39F	CCATGGTGCCATCACCGGC**AAA**ACCATGCAGAACCAGATC
f_hheG_T39C	GATCTGGTTCTGCATGGT**TGC**GCCGGTGATGGCACCATG
r_hheG_T39C	CATGGTGCCATCACCGGC**GCA**ACCATGCAGAACCAGATC
f_hheG_A40K	GATCTGGTTCTGCATGGTACA**AAA**GGTGATGGCACCATGGTTGG
r_hheG_A40K	CCAACCATGGTGCCATCACC**TTT**TGTACCATGCAGAACCAGATC
f_hheG_A40E	GGTTCTGCATGGTACA**GAA**GGTGATGGCACCATGG
r_hheG_A40E	CCATGGTGCCATCACC**TTC**TGTACCATGCAGAACC
f_hheG_A40F	GATCTGGTTCTGCATGGTACA**TTT**GGTGATGGCACCATGGTTGG
r_hheG_A40F	CCAACCATGGTGCCATCACC**AAA**TGTACCATGCAGAACCAGATC
f_hheG_A40C	GGTTCTGCATGGTACA**TGC**GGTGATGGCACCATG
r_hheG_A40C	CATGGTGCCATCACC**GCA**TGTACCATGCAGAACC
f_hheG_G41K	GTTCTGCATGGTACAGCC**AAA**GATGGCACCATGGTTGGTG
r_hheG_G41K	CACCAACCATGGTGCCATC**TTT**GGCTGTACCATGCAGAAC
f_hheG_G41E	CTGCATGGTACAGCC**GAA**GATGGCACCATGGTTG
r_hheG_G41E	CAACCATGGTGCCATC**TTC**GGCTGTACCATGCAG
f_hheG_G41F	GTTCTGCATGGTACAGCC**TTT**GATGGCACCATGGTTG
r_hheG_G41F	CAACCATGGTGCCATC**AAA**GGCTGTACCATGCAGAAC
f_hheG_G41C	GTTCTGCATGGTACAGCC**TGC**GATGGCACCATGGTTGG
r_hheG_G41C	CCAACCATGGTGCCATC**GCA**GGCTGTACCATGCAGAAC
f_hheG_D42K	CTGCATGGTACAGCCGGT**AAA**GGCACCATGGTTGGTGTTG
r_hheG_D42K	CAACACCAACCATGGTGCC**TTT**ACCGGCTGTACCATGCAG
f_hheG_D42E	CATGGTACAGCCGGT**GAA**GGCACCATGGTTGGTG
r_hheG_D42E	CACCAACCATGGTGCC**TTC**ACCGGCTGTACCATG
f_hheG_D42F	GCATGGTACAGCCGGT**TTT**GGCACCATGGTTGG
r_hheG_D42F	CCAACCATGGTGCC**AAA**ACCGGCTGTACCATGC
f_hheG_D42C	CTGCATGGTACAGCCGGT**TGC**GGCACCATGGTTGGTGTTG
r_hheG_D42C	CAACACCAACCATGGTGCC**GCA**ACCGGCTGTACCATGCAG
f_hheG_G43K	GCATGGTACAGCCGGTGAT**AAA**ACCATGGTTGGTGTTGAAG
r_hheG_G43K	CTTCAACACCAACCATGGT**TTT**ATCACCGGCTGTACCATGC
f_hheG_G43E	CATGGTACAGCCGGTGAT**GAA**ACCATGGTTGGTGTTGAAG
r_hheG_G43E	CTTCAACACCAACCATGGT**TTC**ATCACCGGCTGTACCATG
f_hheG_G43F	GCATGGTACAGCCGGTGAT**TTT**ACCATGGTTGGTGTTGAAG
r_hheG_G43F	CTTCAACACCAACCATGGT**AAA**ATCACCGGCTGTACCATGC
f_hheG_G43C	CATGGTACAGCCGGTGAT**TGC**ACCATGGTTGGTGTTG
r_hheG_G43C	CAACACCAACCATGGT**GCA**ATCACCGGCTGTACCATG
f_hheG_T44K	GGTACAGCCGGTGATGGC**AAA**ATGGTTGGTGTTGAAGAAAG
r_hheG_T44K	CTTTCTTCAACACCAACCAT**TTT**GCCATCACCGGCTGTACC
f_hheG_T44E	CATGGTACAGCCGGTGATGGC**GAA**ATGGTTGGTGTTGAAGAAAG
r_hheG_T44E	CTTTCTTCAACACCAACCAT**TTC**GCCATCACCGGCTGTACCATG

f_hheG_T44F	CATGGTACAGCCGGTGATGGC**TTT**ATGGTTGGTGTTGAAGAAAG
r_hheG_T44F	CTTTCTTCAACACCAACCAT**AAA**GCCATCACCGGCTGTACCATG
f_hheG_T44C	GTACAGCCGGTGATGGC**TGC**ATGGTTGGTGTTGAAG
r_hheG_T44C	CTTCAACACCAACCAT**GCA**GCCATCACCGGCTGTAC
f_hheG_M45E	GGTACAGCCGGTGATGGCACC**GAA**GTTGGTGTTGAAGAAAGTTTTG
r_hheG_M45E	CAAAACTTTCTTCAACACCAAC**TTC**GGTGCCATCACCGGCTGTACC
f_hheG_M45F	GGTACAGCCGGTGATGGCACC**TTT**GTTGGTGTTGAAGAAAGTTTTG
r_hheG_M45F	CAAAACTTTCTTCAACACCAAC**AAA**GGTGCCATCACCGGCTGTACC
f_hheG_V46E	CCGGTGATGGCACCATG**GAA**GGTGTTGAAGAAAGTTTTG
r_hheG_V46E	CAAAACTTTCTTCAACACC**TTC**CATGGTGCCATCACCGG
f_hheG_V46F	CCGGTGATGGCACCATG**TTT**GGTGTTGAAGAAAG
r_hheG_V46F	CTTTCTTCAACACC**AAA**CATGGTGCCATCACCGG
f_hheG_V46C	GCCGGTGATGGCACCATG**TGC**GGTGTTGAAGAAAGTTTTG
r_hheG_V46C	CAAAACTTTCTTCAACACC**GCA**CATGGTGCCATCACCGGC
f_hheG_G47K	GCCGGTGATGGCACCATGGTT**AAA**GTTGAAGAAAGTTTTGATAGCC
r_hheG_G47K	GGCTATCAAAACTTTCTTCAAC**TTT**AACCATGGTGCCATCACCGGC
f_hheG_G47E	CGGTGATGGCACCATGGTT**GAA**GTTGAAGAAAGTTTTGATAG
r_hheG_G47E	CTATCAAAACTTTCTTCAAC**TTC**AACCATGGTGCCATCACCG
f_hheG_G47F	CCGGTGATGGCACCATGGTT**TTT**GTTGAAGAAAGTTTTGATAG
r_hheG_G47F	CTATCAAAACTTTCTTCAAC**AAA**AACCATGGTGCCATCACCGG
f_hheG_G47C	CCGGTGATGGCACCATGGTT**TGC**GTTGAAGAAAGTTTTGATAG
r_hheG_G47C	CTATCAAAACTTTCTTCAAC**GCA**AACCATGGTGCCATCACCGG
r_hheG_T44-46	GGTACAGCCGGTGATGGCAGAGACCA
f_hheG_M45K	AGGTCTCATGGCACC**AAA**GTTGGTGTTGAAGAAAGTTTTGATAGCC
f_T44V	AGGTCTCATGGC**GTT**ATGGTTGGTGTTGAA GAAAGTTTTGATAGCC
f_M45V	AGGTCTCATGGCACC**GTT**GTTGGTGTTGAAGAAAGTTTTGATAGCC
f_T44L	AGGTCTCATGGC**CTG**MTGGTTGGTGTTGAAGAAAGTTTTGATAGCC
f_M45L	AGGTCTCATGGCACC**CTG**GTTGGTGTTGAAGAAAGTTTTGATAGCC
f_V46L	AGGTCTCATGGCACCATG**CTG**GGTGTTGAAGAAAGTTTTGATAGCC
f_T44I	AGGTCTCATGGC**ATT**ATGGTTGGTGTTGAAGAAAGTTTTG ATAGCC
f_M45I	AGGTCTCATGGCACC**ATT**GTTGGTGTTGAAGAAAGTTTTGATAGCC
f_V46I	AGGTCTCATGGCACCATG**ATT**GGTGTTGAAGAAAGTTTTG ATAGCC
f_T44M	AGGTCTCATGGC**ATG**ATGGTTGGTGTTGAAGAAAGTTTTGATAGCC
f_V46M	AGGTCTCATGGCACCATG**ATG**GGTGTTGAAGAAAGTTTTG ATAGCC
f_T44D	AGGTCTCATGGC**GAT**ATGGTTGGTGTTGAAGAAAGTTTTGATAGCC
f_M45D	AGGTCTCATGGCACC**GAT**GTTGGTGTTGAAGAAAGTTTTG ATAGCC
f_V46D	AGGTCTCATGGCACCATG**GAT**GGTGTTGAAGAAAGTTTTGATAGCC
f_T44Y	AGGTCTCATGGC**TAT**ATGGTTGGTGTTGAAGAAAGTTTTGATAGCC
f_M45Y	AGGTCTCATGGCACC**TAT**GTTGGTGTTGAAGAAAGTTTTGATAGCC
f_V46Y	AGGTCTCATGGCACCATG**TAT**GGTGTTGAAGAAAGTTTTGATAGCC
f_T44N	AGGTCTCATGGC**AAC**ATGGTTGGTGTTGAAGAAAGTTTTGATAGCC
f_M45N	AGGTCTCATGGCACC**AAC**GTTGGTGTTGAAGAAAGTTTTGATAGCC
f_V46N	AGGTCTCATGGCACCATG**AAC**GGTGTTGAAGAAAGTTTTGATAGCC
f_M45K	AGGTCTCATGGCACC**AAA**GTTGGTGTTGAAGAAAGTTTTGATAGCC
f_T44Q	AGGTCTCATGGC**CAG**ATGGTTGGTGTTGAAGAAAGTTTTGATAGCC
F_M45Q	AGGTCTCATGGCACC**CAG**GTTGGTGTTGAAGAAAGTTTTGATAGCC
f_V46Q	AGGTCTCATGGCACCATG**CAG**GGTGTTGAAGAAAGTTTTGATAGCC
f_T44A	AGGTCTCATGGC**GCG**ATGGTTGGTGTTGAAGAAAGTTTTGATAGCC
f_M45A	AGGTCTCATGGCACC**GCG**GTTGGTGTTGAAGAAAGTTTTGATAGCC
f_V46A	AGGTCTCATGGCACCATG**GCG**GGTGTTGAAGAAAGTTTTGATAGCC
f_T44P	AGGTCTCATGGC**CCG**ATGGTTGGTGTTGAAGAAAGTTTTGATAGCC
f_M45P	AGGTCTCATGGCACC**CCG**GTTGGTGTTGAAGAAAGTTTTGATAGCC
f_V46P	AGGTCTCATGGCACCATG**CCG**GGTGTTGAAGAAAGTTTTGATAGCC
f_T44S	AGGTCTCATGGC**AGC**ATGGTTGGTGTTGAAGAAAGTTTTGATAGCC
f_M45S	AGGTCTCATGGCACC**AGC**GTTGGTGTTGAAGAAAGTTTTGATAGCC
f_V46S	AGGTCTCATGGCACCATG**AGC**GGTGTTGAAGAAAGTTTTGATAGCC
f_M45T	AGGTCTCATGGCACC**ACC**GTTGGTGTTGAAGAAAGTTTTGATAGCC
f_V46T	AGGTCTCATGGCACCATG**ACC**GGTGTTGAAGAAAGTTTTGATAGCC
f_T44G	AGGTCTCATGGC**GGC**ATGGTTGGTGTTGAAGAAAGTTTTGATAGCC
f_M45G	AGGTCTCATGGCACC**GGC**GTTGGTGTTGAAGAAAGTTTTGATAGCC
F_V46G	AGGTCTCATGGCACCATG**GGC**GGTGTTGAAGAAAGTTTTGATAGCC
f_T44W	AGGTCTCATGGC**TGG**ATGGTTGGTGTTGAAGAAAGTTTTGATAGCC
f_M45W	AGGTCTCATGGCACC**TGG**GTTGGTGTTGAAGAAAGTTTTGATAGCC
f_V46W	AGGTCTCATGGCACCATG**TGG**GGTGTTGAAGAAAGTTTTGATAGCC
f_T44R	AGGTCTCATGGC**CGC**ATGGTTGGTGTTGAAGAAAGTTTTGATAGCC

f_M45R	AGGTCTCATGGCACC**CGC**GTTGGTGTTGAAGAAAGTTTTGATAGCC
f_V46R	AGGTCTCATGGCACCATG**CGC**GGTGTTGAAGAAAGTTTTGATAGCC
f_hheG_T44M45V46_del	AGGTCTCATGGCGGTGTTGAAGAAAGTTTTGATAGCCAGATTGC
r_hheG_T44M45V46_del	TGGTCTCTGCCATCACCGGCTGTACC
f_hheG_T39G47_del	AGGTCTCATGGTGTTGAAGAAAGTTTTGATAGCCAGATTGCCG
r_hheG_T39G47_del	TGGTCTCTACCATGCAGAACCAGATCAAAACCACG
f_hheG_T39G47_DPAE	AGGTCTCACGGCCGAAGTTGAAGAAAGTTTTGATAGCCAGATTGCCG
r_hheG_T39G47_DPAE	TGGTCTCTGCCGGATCACCATGCAGAACCAGATCAAAACCACG

Protein production in 100 mL scale and purification via gravity-flow

Selected HheG variants displaying improved enantioselectivity during screening as well as loop3947 deletion variants were produced in shaking flasks containing 100 mL TB medium supplemented with 50 µg mL^{-1} kanamycine and 0.2 mM IPTG. After inoculation with 10% (v/v) pre-culture, protein production was performed for 24 h at 22 °C and 220 rpm. Cells were harvested by centrifugation (3494 *g*, 20 min, 4 °C) and resulting cell pellets were stored at -20 °C until further use.

For subsequent IMAC-based purification using gravity-flow columns, cells were resuspended in 20 mL lysis buffer (50 mM Tris·SO_4 buffer, pH 7.9, 25 mM imidazole) supplemented with 1 mg mL^{-1} lysozyme and 1 pierce protease inhibitor tablet. Sonification was perfomed on ice (65% amplitude, 10 s pulse, 20 s pause). Cell debris was removed by centrifugation (18000 *g*, 45 min, 4 °C) and the resulting CFE was filtered through a 0.45 µm filter. Protein purification was performed using pierce gravity-flow columns (Thermo Fisher Scientific) containing 2 mL Ni-NTA sepharose (Cytiva, Freiburg, Germany). The column material was first equilibrated with 10 column volumes (CV) lysis buffer. The CFE was loaded on the column. After flow through, the column was washed with 10 CV lysis buffer. For elution of desired proteins, 10 CV elution buffer (50 mM Tris·SO_4 buffer, pH 7.9, 500 mM imidazole) were used. The first 2.5 mL of elution containing the desired protein were collected and desalted using PD10 desalting columns in combination with TE buffer (10 mM Tris·SO_4 buffer, pH 7.9, 4 mM ethylenediamine tetraacetic acid, 10% (v/v) glycerol) according to the manufacturer's instructions. Desalted proteins were stored at -20 °C until further use. Protein concentrations were determined by measuring the absorbance at 280 nm using an NP80 nanophotometer (Implen, München, Germany) and calculated via the Lambert-Beer law with molar extinction coefficients and molecular weights of the respective proteins obtained from Protparam[5].

Protein production in 500 mL scale and purification via FPLC

For kinetic analysis of HheG M45F as well as crystallization of HheG M45F-D114C, both variants were produced in 500 mL scale according to the protocol described above, and purified via their N-terminal His-tag using fast protein liquid chromatography (FPLC). Cell disruption was performed as described above but using 30 mL buffer A (50 mM Tris·SO_4, 300 mM Na_2SO_4, 25 mM imidazole, pH 7.9), supplemented with 1 mg mL^{-1} lysozyme and 1 Pierce Protease Inhibitor Mini Tablet, for cell resuspension. Cell free extracts were loaded with a flow rate of 2 mL min^{-1} on a 5 mL HisTrap FF column (GE Healthcare, Freiburg, Germany), pre-equlibrated with lysis buffer, using an ÄktaStart FPLC system (GE Healthcare). Afterwards, the column was washed with 10 CV of buffer A to remove other proteins. His-tagged protein was eluted using a gradient over 60 mL to 100% buffer B (50 mM Tris·SO_4, 300 mM Na_2SO_4, 500 mM imidazole, pH 7.9) while collecting 1 mL fractions. Fractions with highest UV absorbance were pooled and desalted using a HiPrep 26/10 desalting column (GE Healthcare), pre-equilibrated with TE buffer (10 mM Tris·SO_4, 4 mM EDTA, pH 7.9, 10% (v/v) glycerol). During desalting, protein was eluted using TE buffer. Desalted protein fractions with highest UV absorbance were concentrated using Vivaspin Turbo 15 centrifugation units (Sartorius, Göttingen, Germany) with 10 kDa molecular weight cut-off. Protein concentrations of resulting purified protein solutions were determined as described above. Both HheG variants were stored at -20 °C until further use.

Protein crystallization and cross-linking

Crystallization and cross-linking of HheG variant M45F-D114C was performed as described before for variant D114C in 200 µL scale.[6] In a vial, 100 µL of a 24 mg mL^{-1} protein solution were mixed with 100 µL crystallization buffer (10 mM HEPES, pH 7.0, 8% (w/v) PEG4000) and incubated for 24 h at 8 °C. The resulting crystals were collected by centrifugation for 3 min at 400 *g* and cross-linked for 24 h at 8 °C with 2 mM bis-maleimidoethane (ThermoFisher Scientific) dissvoled in crystallization buffer containing 10% (v/v) dimethylsulfoxide. After cross-linking, CLECs were collected by centrifugation,

washed with 200 µL 50 mM Tris·SO_4, pH 7.0, and centrifuged again. Obtained CLECs were resuspended in 50 mM Tris·SO_4 buffer, pH 7.0 to a concentration of 2 mg mL^{-1}. Microscopic analysis of the CLECs was performed using microscope SMZ-171-TLED (Moticeurope, Barcelona, Spain).

Biocatalysis using purified enzymes

Biocatalytic reactions using purified enzymes (HheG wild type and selected variants) were performed under the same reaction conditions as described for library screening but using 5 to 400 µg mL^{-1} of purified enzyme. Samples were taken after specific time points (2 h for epoxide **1** + azide; 24 h for epoxide **1** + cyanide, cyanate and nitrite; 10 min for epoxide **3** + azide; 30 min for epoxide **5** + azide), extracted with an equal volume of TBME containing 0.1% dodecane as internal standard and analyzed by achiral and chiral GC (Table S13). Time points were selected to minimize chemical background reactions. All reactions were performed in duplicate. E values were calculated according to Chen et al.[7]

Reactions to compare soluble HheG wild-type and soluble variant M45F-D114C with CLECs of HheG M45F-D114C were performed in 1 mL 50 mM Tris·SO_4 buffer, pH 7.0 containing 20 mM cyclohexene oxide (**1**), 40 mM azide and 200 µg biocatalyst. Reactions were incubated for 1 h at 22 °C and 900 rpm. Reactions were performed in duplicate, extracted with an equal volume of TBME containing 0.1% dodecane as internal standard and analyzed by achiral and chiral GC (Table S13).

Preparative-scale reaction

A preparative scale reaction using CLECs of HheG M45-D114C was performed in 10 mL 50 mM Tris·SO_4 buffer, pH 7.0 containing 50 mM cyclohexene oxide (**1**), 100 mM azide and 2 mg CLECs. The reaction was stirred for 2 h at room temperature and 800 rpm. Afterwards, the reaction mixture was extracted once with 10 mL TBME, the organic fraction was dried over anhydrous $MgSO_4$ and the solvent was removed by evaporation yielding the product as yellowish oil.[8] The product was further analyzed by achiral and chiral GC (Table S13).

Thermal shift assay

Apparent melting temperatures (T_m) of purified HheG variants were determine by thermal shift assay according to a previously described protocol.[6] Each measurement contained 10 µg biocatalyst and 5x SYPRO orange fluorescent dye in TE buffer in a total of 50 µL volume.

Halide release assay

Specific activities of selected HheG variants (wild type and M45F) in the dehalogenation of 2-chlorocyclohexanol (**2e**) were determined by halide release assay as described previously.[4] Reactions were performed in 1 mL 25 mM Tris·SO_4 buffer, pH 7.0 containing 20 mM **2e** and 400 µg biocatalyst at 22 °C. Samples were taken after 30, 80, 180, 270 and 360 s. Specific activities were calculated based on released halide ions. Reactions were performed in duplicate. Chemical background dehalogenation in reactions without enzyme addition was substracted.

Gas chromatography (GC)

Table S13. GC temperature programs and retention times of substrates and products used in this study.

**Compound**	**Temperature program**	**Retention time**
**Achiral separation (OPTIMA 5 MS)**		
2-chlorocyclohexan-1-ol (**2e**)	7.5 min at 110 °C // heating with 50 °C min^{-1} to 295 °C	3.1 min
cyclohexene oxide (**1**)		2.0 min
2-azidocyclohexan-1-ol (**2a**)		5.4 min
2-cyanocyclohexan-1-ol (**2b**)		5.2 min
2-nitrocyclohexan-1-ol (**2c**)		5.6, 6.4, 7.1 min
hexahydrobenzo[d]oxazol-2(3H)-one (**2d**)		10.1 min
cyclohexan-1,2-diol (**2f**)		3.4 min
trans-2,3-heptene oxide (**5**)	2 min at 70 °C // heating with 50°C min^{-1} to 125 °C // 3 min at 125 °C // heating with 50 °C min^{-1} to 300 °C	3.0 min
2-azidoheptan-3-ol (**6a**)		6.1 min
3-azidoheptan-2-ol (**6b**)		6.0 min
**Chiral separation (HYDRODEX γ-DIMOM)**		
cyclohexene oxide (**1**)	40 min at 100°C // heating with 10 °C min^{-1} to 200 °C	3.1 min
(1*S*,2*S*)-2-azidocyclohexan-1-ol (**2a**)		34.1 min
(1*R*,2*R*)-2-azidocyclohexan-1-ol (**2a**)		35.4 min
cyclohexene oxide (**1**)	Heating from 40 to 90 °C with 10 °C min^{-1} // 20 min at 90 °C // heating with 5 °C min^{-1} to 220 °C	7.7 min
(1*S*,2*R*)-2-cyanocyclohexan-1-ol (**2b**)		40.0 min
(1*R*,2*S*)-2-cyanocyclohexan-1-ol (**2b**)		40.2 min
rac-2-nitrocyclohexan-1-ol (**2c**)		40.6, 41.3 min
rac-hexahydrobenzo[d]oxazol-2(3H)-one (**2d**)		48.8, 49.1 min
(*R*)-styrene oxide (**3**)	15 min at 90 °C // heating with 10 °C min^{-1} to 140 °C // 10 min at 140 °C // heating with 0,5 °C min^{-1} to 150 °C // 10 min at 150 °C // heating with 10 °C min^{-1} to 200 °C, hold for 1 min	11.0 min
(*S*)-styrene oxide (**3**)		11.6 min
(R)-2-azidophenylethan-1-ol (**4**)		33.1 min
(S)-2-azidophenylethan-1-ol (**4**)		33.5 min
**Chiral separation (Lipodex E)**		
(2*R,3S*)-2-azidoheptan-3-ol (**6a**)	Heating from 85 to 95 °C with 0,5 °C min^{-1} // heating with 10 °C min^{-1} to 200 °C	17.5 min
(2*S,3R*)-2-azidoheptan-3-ol (**6a**)		17.9 min
(2*S,3R*)-3-azidoheptan-2-ol (**6b**)		19.6 min
(2*R,3S*)-3-azidoheptan-2-ol (**6b**)		20.7 min

References

(1) Staar, S.; Estévez-Gay, M.; Kaspar, F.; Osuna, S.; Schallmey, A. Engineering of Conserved Sequence Motif 1 Residues in Halohydrin Dehalogenase HheC Simultaneously Enhances Activity, Stability and Enantioselectivity. **2024**. https://doi.org/10.26434/chemrxiv-2024-cw4cm.

(2) Boto, R. A.; Peccati, F.; Laplaza, R.; Quan, C.; Carbone, A.; Piquemal, J.-P.; Maday, Y.; Contreras-García, J. NCIPLOT4: Fast, Robust, and Quantitative Analysis of Noncovalent Interactions. *J. Chem. Theory Comput.* **2020**, *16* (7), 4150–4158. https://doi.org/10.1021/acs.jctc.0c00063.

(3) Calderini, E.; Wessel, J.; Süss, P.; Schrepfer, P.; Wardenga, R.; Schallmey, A. Selective Ring-Opening of Di-Substituted Epoxides Catalysed by Halohydrin Dehalogenases. *ChemCatChem* **2019**, *11* (8), 2099–2106. https://doi.org/10.1002/cctc.201900103.

(4) Koopmeiners, J.; Halmschlag, B.; Schallmey, M.; Schallmey, A. Biochemical and Biocatalytic Characterization of 17 Novel Halohydrin Dehalogenases. *Appl. Microbiol. Biotechnol.* **2016**, *100* (17), 7517–7527. https://doi.org/10.1007/s00253-016-7493-9.

(5) Gasteiger, E.; Hoogland, C.; Gattiker, A.; Duvaud, S.; Wilkins, M. R.; Appel, R. D.; Bairoch, A. Protein Identification and Analysis Tools on the ExPASy Server. In *The Proteomics Protocols Handbook*; Walker, J. M., Ed.; Springer Protocols Handbooks; Humana Press: Totowa, NJ, 2005; pp 571–607. https://doi.org/10.1385/1-59259-890-0:571.

(6) Staar, M.; Henke, S.; Blankenfeldt, W.; Schallmey, A. Biocatalytically Active and Stable Cross-Linked Enzyme Crystals of Halohydrin Dehalogenase HheG by Protein Engineering. *ChemCatChem* **2022**, *14* (9), e202200145. https://doi.org/10.1002/cctc.202200145.

(7) Chen, C. S.; Fujimoto, Y.; Girdaukas, G.; Sih, C. J. Quantitative Analyses of Biochemical Kinetic Resolutions of Enantiomers. *J. Am. Chem. Soc.* **1982**, *104* (25), 7294–7299. https://doi.org/10.1021/ja00389a064.

(8) Christoffers, J.; Schulze, Y.; Pickardt, J. Synthesis, Resolution, and Absolute Configuration of Trans-1-Amino-2-Dimethylaminocyclohexane. *Tetrahedron* **2001**, *57* (9), 1765–1769. https://doi.org/10.1016/S0040-4020(00)01172-8.
